# IL-33/NF-κB/ST2L/Rab37 positive-feedback loop promotes M2 macrophage to limit chemotherapeutic efficacy in lung cancer

**DOI:** 10.1038/s41419-024-06746-y

**Published:** 2024-05-22

**Authors:** You-En Yang, Meng-Hsuan Hu, Yen-Chen Zeng, Yau-Lin Tseng, Ying-Yuan Chen, Wu-Chou Su, Chih-Peng Chang, Yi-Ching Wang

**Affiliations:** 1 Institute of Basic Medical Sciences, College of Medicine, National Cheng Kung University, No.1, University Road, Tainan, 701 Taiwan; 2 Department of Pharmacology, College of Medicine, National Cheng Kung University, No.1, University Road, Tainan, 701 Taiwan; 3 Department of Microbiology and Immunology, College of Medicine, National Cheng Kung University, No.1, University Road, Tainan, 701 Taiwan; 4 Division of Thoracic Surgery, Department of Surgery, College of Medicine, National Cheng Kung University, No.1, University Road, Tainan, 701 Taiwan; 5 Division of Oncology, Department of Internal Medicine, College of Medicine, National Cheng Kung University, No.1, University Road, Tainan, 701 Taiwan

**Keywords:** Mechanisms of disease, Cancer microenvironment, Transcription, Small GTPases, Cancer therapy

## Abstract

IL-33 is a danger signal that binds to its receptor ST2L to promote tumor progression. This study identifies the IL-33/ST2L positive-feedback loop and the trafficking of ST2L membrane presentation in macrophages that contribute to lung tumor progression. Mechanistically, IL-33 induces ST2L upregulation by activating NF-κB, which binds to the promoter region of the *ST2L* gene. Moreover, Rab37, a small GTPase involved in membrane trafficking, mediates ST2L trafficking to the plasma membrane of M2 macrophages. This IL-33/NF-κB/ST2L/Rab37 axis promotes positive-feedback loops that enhance ST2L expression and membrane trafficking in M2 macrophages. Notably, neutralizing antibodies against IL-33 or ST2L block NF-κB activity, suppress M2 macrophage polarization, and synergistically inhibit tumor growth when combined with cisplatin treatment in vitro/vivo. Clinically, Rab37^+^/ST2L^+^/CD206^+^ tumor-infiltrating M2 macrophages correlate with advanced-stage lung cancer patients with poor response to chemotherapy. These findings unveil a positive-feedback mechanism and provide a basis for IL-33/ST2L-targeting therapy for cancer.

## Introduction

Interleukin-33 (IL-33), a member of the IL-1 family, mediates its biological effects *via* binding to the transmembrane receptor, a longer isoform of suppression of tumorigenicity 2 (ST2L) [1]. Upon tissue stress or damage, IL-33 is quickly released from the nucleus of necrotic cells to function as a damage-associated molecular pattern (DAMP) molecule that provides a ‘danger’ signal by activating cells of lymphoid and myeloid origin [2]. The IL-33/ST2L pathway influences tissue homeostasis, infection responses, and inflammation [3, 4]. Recently, many reports have drawn attention from the roles of IL-33/ST2L to their effects in facilitating communication between tumor cells and immune cells within the tumor microenvironment (TME), significantly influencing the remodeling of immunosuppressive TME [5–8]. The IL-33/ST2L pathway, associated with type 2 immunity via ST2L^+^ regulatory T cells (Tregs) activation, leads to increased Treg accumulation in the TME, promoting poor prognosis in various cancers [9, 10]. In addition, IL-33 secretion in the tumor sites induces M2-like macrophage differentiation and favors tumor growth and tumor metastasis in esophageal cancer [11]. However, the mechanism of IL-33/ST2L-induced M2 macrophage polarization in macrophages remains elusive. In addition, the process of ST2L membrane trafficking is still not understood.

The Rab family of small GTPases functions as crucial regulators of membrane trafficking and protein transport. Our previous studies demonstrate that Rab37 is involved in the exocytosis of secretory and membrane proteins in both non-small-cell lung cancer (NSCLC) and immune cells like macrophages and T cells [12–14]. Notably, the Rab37-mediated secretory mechanism of the soluble isoform of ST (sST2) has been revealed in NSCLC [15]. Therefore, we hypothesized that Rab37 also mediates trafficking and membrane presentation of ST2L in macrophages.

Recent evidence indicates a correlation between tumor-associated macrophages (TAMs) and chemotherapy efficacy [16, 17]. In lung cancer, cisplatin resistance enhances self-renewal and induces M2 polarization of TAMs through the secretion of macrophage migration inhibitory factor [18]. Additionally, many studies have shown that chemotherapy treatment induces chronic inflammation and increases M2 macrophages in TME by upregulating DAMPs [19, 20]. Nevertheless, the potential impact of IL-33 as a DAMP in modulating the composition of the TME and thereby affecting the therapeutic efficacy of cisplatin remains unexplored.

Here, we identify the cisplatin-induced danger signal IL-33, which promotes M2 macrophages to reduce the anti-tumor effect of cisplatin in lung cancer. Mechanistically, IL-33 enhances *ST2L* mRNA expression in M2 macrophages *via* positive-feedback regulation of IL-33/NF-κB/ST2L axis. Subsequently, ST2L protein is transported to the plasma membrane by Rab37-mediated membrane trafficking to serve as the receptor of IL-33. This positive transcription and membrane trafficking loop amplifies IL-33-mediated M2 polarization, increasing cisplatin resistance. The combination of cisplatin and neutralizing antibodies against IL-33 or ST2L alleviates cisplatin resistance by significantly suppressing lung tumor growth in the allograft mouse models. Clinically, late-stage NSCLC patients with increased intratumoral Rab37^+^ST2^+^CD206^+^ M2-TAMs show poor prognosis. Our findings highlight the IL-33/ST2L axis as a potential target for overcoming cisplatin resistance in NSCLC patients.

## Materials and methods

### Mouse, cell lines, culture conditions, and plasmid

Bone marrow cells were aseptically harvested from hind legs of 6-to-8 week-old wild-type (WT) or *Rab37* knockout (KO) C57BL/6 mice [13]. They were cultured in macrophage medium with DMEM containing 10% Fetal Bovine Serum (FBS, Gibco, Waltham, MA, US), 1% penicillin/streptomycin (Gibco), and 10 ng/mL macrophage colony-stimulating factor (M-CSF) (#315-02, PeproTech, Cranbury, NJ, USA)) at 37 °C, 5% CO_2_ for 7 days to differentiate into bone marrow-derived macrophages (BMDMs).

THP-1, RAW264.7, A549, and Lewis lung carcinoma (LLC) were purchased from the American Type Culture Collection. THP-1 was maintained in RPMI (Gibco), while RAW264.7, A549, and LLC were in DMEM (Gibco) with 10% FBS (Gibco) and 1% penicillin/streptomycin (Gibco), and cultured at 37 ^o^C with 5% CO_2_ in air.

cDNA of mouse Rab37 was purchased from OriGene and its mutants (Rab37-Q89L, Rab37-T43N) were PCR-amplified and inserted into the pcDNA-His-V5 vector (Invitrogen, Waltham, Massachusetts, USA). RFP-tagged Rab37 was created by cloning Rab37 cDNA into the pDsRed2-C1 vector (NovoPro Bioscience Inc., Shanghai, China). Plasmid pLV-mIL1RL1-GFPSpark (ST2L expression vector) was purchased from Sino Biological Inc (Beijing, China). Plasmids used in the study are listed in Supplementary Table S1. The *ST2L* promoter region (−1549 to +82 of the transcriptional start site, TSS) was cloned into the pGL4.17 luciferase vector. Site-directed mutagenesis was used to mutate the p65-motif at two sites in the *ST2L* promoter, utilizing specific primers listed in Supplementary Table S2.

### Membrane fractionation

A total of 1 × 10^7^ WT, *Rab37* KO BMDMs and RAW264.7 cells expressing EV, Rab37-WT, Rab37-QL, or Rab37-TN, were treated with 20 ng/mL recombinant IL-13 (rIL-13, #210-13, PeproTech) for 24 h, followed by treatment with novel cell-permeable clathrin inhibitor Pitstop® 2 (#ab120687, Abcam, Cambridge, UK) 20 µM 10 min in serum-free medium and then with rIL-33 (#210-33, PeproTech) 50 ng/mL for 15 min in serum-free medium. The membrane fractionation was performed according to the manufacturer’s instructions (#444810, Merck Millipore, Darmstadt, Germany).

### Vesicle isolation and immunoprecipitation (IP)

Vesicle isolation protocol was modified from Hendrix’s report [21]. RAW264.7 cells were sonicated and supernatants were obtained by centrifugation (3000 × *g* for 10 min at 4 °C). Vesicles were enriched from the resulting supernatants by high-speed centrifugation (30,000 × *g* for 60 min at 4 °C) using a 40-Ti rotor (Beckman Coulter, Brea, CA, USA). The vesicle-containing solution was incubated with anti-V5 antibody to isolate Rab37-specific vesicles.

### Conditioned medium (CM) preparation, collection and treatment

LLC and A549 cells were cultured and treated with various concentrations of cisplatin for 24 h. The CM was then harvested, cleared by centrifugation, and stored. In addition, CM-treated RAW264.7 and THP-1 cells were then harvested after 24 h. In some experiments, α-IL-33 (#LGT3314, Leadgene Biomedical, Tainan, Taiwan) and α-ST2L (#LGT2105, Leadgene Biomedical) were added to the CM at a concentration of 1 μg/ml or 5 μg/ml.

### Chromatin immunoprecipitation assay (ChIP) assay

Differentiated THP-1 macrophages were treated with IL-33 (#200-33, PeproTech) 100 ng/mL, IL-13 (#200-13, PeproTech) 20 ng/mL, and IL-33 + IL-13 for 24 h. The cells were then harvested and cross-linked, followed by preparation of nuclear lysates using the Magna ChIP^TM^ protein G Kit (Merck Millipore). Nuclear lysates were sonicated to shear DNA to around 500 bp using QSONICA sonicator with cooling system (on 45 s, off 30 s, 40 cycles) followed by immunoprecipitation with anti-p-65 (Ser536) antibody (#3033, Cell Signaling, Danvers, MA, US). q-PCR was carried out using ChIP products. Results were normalized with input control. The primer conditions are described in Supplementary Table S2.

### Dual Luciferase promoter activity assay

RAW264.7 cells were seeded in 12-well plates before transfection. After 6 h of co-transfection with either an empty vector, WT, or mutated gene promoter vector, cells were treated with IL-13 (20 ng/mL), or IL-13 (20 ng/mL) + IL-33 (100 ng/mL) for 24 h. The dual luciferase promoter assay kit (Promega, Madison, WI, USA) was used to determine gene promoter activity according to the manufacturer’s protocols.

### RAW-Blue^TM^ cells (NF-κB activation) assay

For the α-IL-33 antibody group, rIL-33 (100 ng/mL) was incubated with α-IL-33 antibody in DMEM medium at 37 °C for 1 h. The solution was then used to treat RAW-Blue cells at 37 °C for 24 h. For the α-ST2L antibody group, RAW-Blue cells were treated with α-ST2L antibody in DMEM medium at 37 °C for 1 h. After 1 h, rIL-33 100 ng/mL was added to the RAW-Blue cells and incubated at 37 °C for 24 h. NF-κB activation was measured using a RAW-Blue^TM^ cells kit (InvivoGen, San Diego, CA, USA) following the manufacturer’s instructions.

### Flow cytometry analysis

BMDMs, THP-1, and RAW264.7 were harvested, washed, and re-suspended in staining buffer (2% FBS, 0.1% sodium azide in PBS) and then stained with anti-ST2L, anti-CD11b, and anti-CD206 antibodies (BD Biosciences, San Jose, CA, USA).

To assess IL-33 binding to the ST2L receptor, RAW264.7 cells were mixed with His-tagged rIL-33 (Leadgene Biomedical) (100 ng/mL) and α-IL-33 (Leadgene Biomedical) or α-ST2L (Leadgene Biomedical) (1 or 5 μg/mL) for 1 h, followed by incubation with a FITC-labeled His Tag antibody and flow cytometry analysis.

Tumor tissues were digested with 0.1 mg/mL collagenase (Sigma-Aldrich, Saint Louis, MO, USA) and 1 mg/mL dispase II (Sigma-Aldrich) in serum-free DMEM for 30 min at 37 °C and meshed with complete medium. Cells were stained with anti-mouse CD11b, CD206, ST2L, and CD86 (BD Bioscience) in addition to CD4, CD25, and Foxp3. The data were recorded by CytoFLEX (Beckman Coulter). The detailed antibody conditions are listed in Supplementary Table S3.

### Allograft tumor mouse model

All animal experiments were performed in compliance with NCKU institutional guidelines for use and care of animals (Permit Numbers: # 110091). For α-IL-33 and α-ST2L antibody treatment, the LLC subcutaneous injection model was used. A total of 1 × 10^6^ LLC cells were subcutaneously injected into flank of C57BL/6 mice. Mice harboring LLC allografts were treated with IgG (10 mg/kg), cisplatin (1 mg/kg), α-IL-33 (10 mg/kg), and/or α-ST2L (10 mg/kg) *via* intraperitoneal injection (i.p.). Cisplatin was administered intraperitoneally (i.p.) on day 7 after inoculation, followed by doses every 3 days, four times in total. For α-IL-33 or α-ST2L groups, treatments were given i.p. on days 13 and 16 post-inoculation, both for mono-treatment and combination therapies. Tumor volume was calculated on day 7 and once every 3 days using the equation V = (a^2^ × b)/2 during observation.

### Multiplex fluorescence immunohistochemistry (IF-IHC)

IF-IHC was performed to detect the percentage of CD206, Rab37, and ST2L co-localization in tumor specimens from 48 lung cancer patients, using the Opal stain kit (#NEL810001KT, Akoya Biosciences, Marlborough, MA, USA) and following the manufacturer’s protocol. Quantitative of IF-IHC was defined by an average of immunoreactive positive cells per three ROIs, 80 × 80 μm. A 0.6% cut-off, based on the mean average of patient data, was set for correlation analysis. Images were captured and analyzed for immunoreactivity and co-localization using the Olympus FV3000 confocal microscope with FV31S-SW software (Olympus). The antibodies are listed in Supplementary Table S3.

### Patient samples and clinical information

A total of 48 lung cancer patients from NCKU Hospital were enrolled with institutional review board permission approval (#A-ER-111-517) and patient consent. Overall survival was calculated as the time from the day of surgery to the date of death or the last follow-up. Disease-free survival was calculated as the time from the day of surgery to either the date of disease recurrence or the date of death. Treatment response was assessed with CT scans post-cisplatin and docetaxel treatment, comparing tumor sizes pre- and post-chemotherapy. Partial response was defined as a post-chemotherapy tumor size reduction of over 30%, while a size increase of over 20% indicated progressive disease [22]. Tumor type and disease staging were performed according to the World Health Organization classification and the TNM classification system, respectively.

### Statistical analysis

Cell studies were done in triplicate, except as noted. Animal study group sizes varied by experiment. Statistical analysis included two-tailed Student’s *t* tests for mean ± SD data, Pearson χ2 for protein expression correlation in patients, Kaplan–Meier for survival curves with log-rank tests, and Cox regression for patient outcome risk analysis. The level of statistical significance was taken as *p* value, *, *p* < 0.05; **, *p* < 0.01; ***, *p* < 0.001.

## Results

### mRNA expression of *IL-33* and *ST2L* is increased in M2 macrophages

**Fig. 1:**
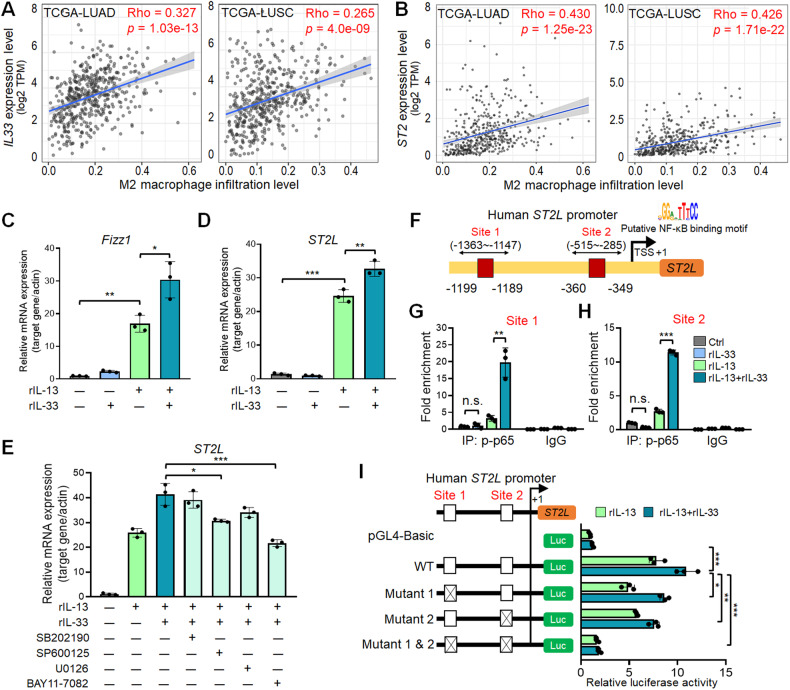
IL-33 promotes M2 macrophage polarization through the IL-33/ST2L/NF-κB/ST2L axis. Correlation analysis of *IL-33* mRNA expression level (**A**) or *ST2* mRNA expression level (**B**) with M2 macrophage infiltration in LUAD (*n* = 515) and LUSC (*n* = 501) using the TIMER2.0 database. RT-qPCR analysis of *Fizz1* (**C**) and *ST2L* (**D**) mRNA expression in THP-1 macrophages treated with rIL-33 (100 ng/mL) and/or rIL-13, 20 ng/mL for 48 h. **E** qPCR analysis of *ST2L* mRNA expression in THP-1 macrophages treated with p38 inhibitor (SB202190, 0.5 mM), JNK inhibitor (SP600125, 0.5 mM), MEK inhibitor (U0126, 0.5 mM) or NF-κB inhibitor (BAY11-7082, 1 mM) during rIL-33 (100 ng/mL) and/or rIL-13, 20 ng/mL treatment. **F** Predicted NF-κB binding sites at the ST2L promoter by PROMO website. **G, H** ChIP-qPCR analysis of p-p65 binding to the *ST2L* promoter in M2 macrophages treated with or without rIL-33 (100 ng/mL) for 24 h. **I** Luciferase reporter assay of WT *ST2L* promoter or mutant *ST2L* promoter with base substitution at the NF-κB motif. Data are presented as mean ± SD, *p < 0.05, **p < 0.01, ***p < 0.001, Student’s *t* test.

Previous studies indicated that IL-33 induces airway inflammation by promoting M2 phenotype in ST2^+^ alveolar macrophages [23, 24]. To explore the potential role of ST2^+^ macrophages in lung cancer, we analyzed the correlation between *IL-33* and *ST2* mRNA levels and macrophage infiltration in lung adenocarcinoma (LUAD) and lung squamous cell carcinoma (LUSC) using TCGA datasets. Our analysis revealed a significant positive correlation between *IL-33* and *ST2* mRNA levels and M2 macrophage infiltration in both LUAD and LUSC datasets, without correlation to M1 macrophage infiltration (Fig. 1A, B and Supplementary Fig. S1A, B). Notably, the transmembrane ST2L and soluble ST2 (sST2) serve as receptor and decoy receptor for IL-33, respectively [1, 25]. To determine which isoform is expressed in lung cancer cells and macrophages, we designed primers targeting two major transcripts derived from alternative splicing: the transmembrane long form *ST2L* (V1) and alternative splicing isoform *sST2* (V2) (Supplementary Fig. S1C). RT-qPCR data revealed *sST2* expression in Lewis lung carcinoma (LLC) (Supplementary Fig. S1D) and elevated *ST2L* expression in RAW264.7 macrophage cells (Supplementary Fig. S1E). Moreover, *ST2L* mRNA levels increased with M2 stimulation and decreased with M1 stimulation in RAW264.7 cells (Supplementary Fig. S1F–H). These findings prompt further investigation into the mechanism of M2 macrophage polarization by IL-33/ST2L signal.

### IL-33 enhances *ST2L* mRNA expression in IL-13-induced M2 macrophages

We first treated recombinant IL-13 (rIL-13) stimulated macrophages with recombinant IL-33 (rIL-33) to assess macrophage polarization. Surprisingly, RT-qPCR analysis revealed that rIL-33 alone did not induce M2 polarization in THP-1, RAW264.7, and bone marrow-derived macrophages (BMDMs) (Fig. 1C and Supplementary Fig. S1I, K). However, rIL-33 further enhanced M2 polarization in rIL-13-treated macrophages when compared to those treated with rIL-13 alone (Fig. 1C and Supplementary Fig. S1I, K). Importantly, the *ST2L* mRNA level increased with rIL-13 treatment and further enhanced upon rIL-33 treatment in THP-1, RAW264.7, and BMDMs (Fig. 1C and Supplementary Fig. S1J, L). Together, these results suggested that increased *ST2L* mRNA expression mediates IL-33-induced M2 polarization in macrophages.

### IL-33/ST2L/NF-κB pathway transcriptionally activates *ST2L* mRNA expression in macrophages to promote M2 polarization

To further understand the mechanism of IL-33-enhanced *ST2L* mRNA expression in rIL-13-induced M2 macrophages, we examined which of the small-molecule inhibitors that target IL-33/ST2L downstream signaling pathways, including p38 (SB202190), JNK (SP600125), MEK (U0126), and NF-κB (BAY11-7082) [26] could inhibit IL-33-enhanced *ST2L* mRNA expression in rIL-13-induced M2 macrophages. RT-qPCR results demonstrated that NF-κB inhibitor most effectively reduced *ST2L* mRNA expression in M2 macrophages induced by both rIL-33 and rIL-13 (Fig. 1E). Furthermore, the overexpression of p65 (NF-κB subunit) further enhanced the *ST2L* mRNA expression induced by IL-13 and IL-33 in macrophages (Supplementary Fig. S1M, N). Conversely, knockdown of p65 significantly inhibited the *ST2L* mRNA expression induced by IL-13 and IL-33 (Supplementary Fig. S1O, P). These findings support the importance of the IL-33/ST2L/NF-κB pathway in regulating *ST2L* mRNA expression in M2 macrophages.

Using the PROMO transcription factor database, we identified two NF-κB binding sites in the *ST2L* promoter (Fig. 1F), leading us to hypothesize that the IL-33/ST2L/NF-κB pathway transcriptionally activates ST2L, promoting a positive-feedback loop in M2 polarization. Chromatin-immunoprecipitation (ChIP)-qPCR results indicated a significant increase in p-p65 binding to the two predicted regions on the *ST2L* promoter in macrophages treated with rIL-13 and rIL-33 (Fig. 1G, H). Next, we performed luciferase reporter assay with wild-type (WT)-*ST2L* promoter or with promoter mutated at −1199~−1189 (Mutant 1), −360~−349 (Mutant 2), or both regions (Mutant 1 & 2). Luciferase reporter assay showed that WT-*ST2L* promoter was induced in macrophages treated with rIL-13, and was further enhanced by rIL-33 (light blue *vs*. dark blue, Fig. 1I). Importantly, IL33-induced *ST2L* promoter activity diminished in Mutant 1 or 2, with base substitution at the NFκB motif, and was completely abolished in the double mutant (Fig. 1I). Collectively, these results demonstrated that IL-33 induces *ST2L* transcription *via* a positive-feedback loop (IL-33/ST2L/NF-κB/ST2L), sustaining IL-13-induced M2 macrophages.

### Knockout of Rab37 decreases ST2L membrane presentation in M2 macrophages

**Fig. 2:**
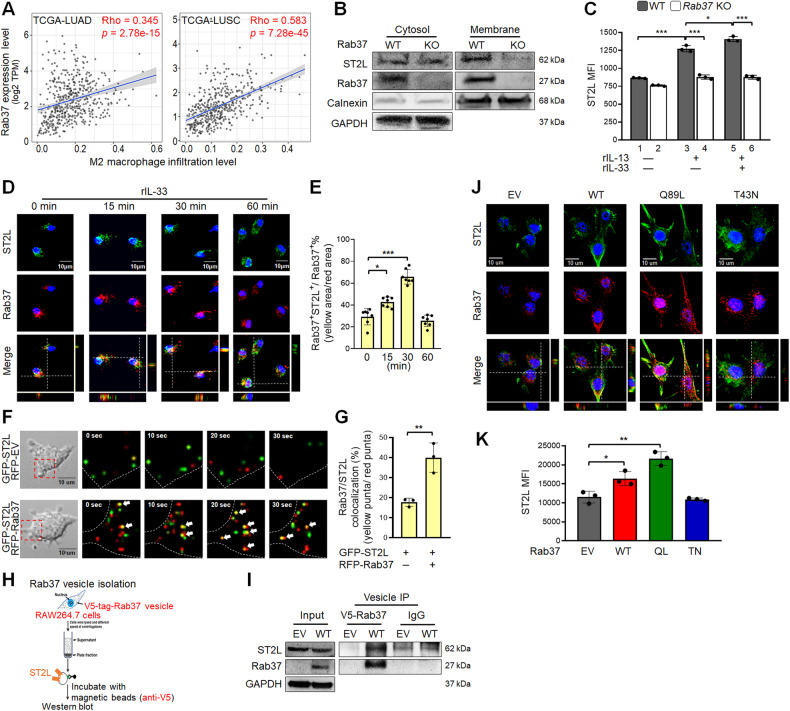
Rab37-mediated ST2L membrane presentation in macrophages in a GTP-dependent manner. **A** Correlation analysis of *Rab37* mRNA expression level with M2 macrophage infiltration in LUAD (*n* = 515) and LUSC (*n* = 501) using the TIMER2.0 database. **B** Comparison of ST2L membrane presentation in WT and *Rab37* knockout (KO) bone marrow-derived macrophages (BMDMs) treated with rIL-13 (20 ng/mL) for 48 h, followed by treatment with rIL-33 (50 ng/mL) for 30 min, by immunoblotting of membrane fraction analysis. **C** Flow cytometry analysis of ST2L membrane expression in WT and *Rab37* KO BMDMs after treatment with rIL-13 (20 ng/mL) for 48 h, followed by treatment with rIL-33 (50 ng/mL) for 30 minutes. **D**, **E** Confocal IF analysis of the localization of Rab37 (red) and ST2L (green) in WT BMDMs treated with rIL-33 (50 ng/mL) at 0, 15, 30 and 60 min (**D**). The percentage of Rab37^+^ST2L^+^ (yellow) and quantification data (**E**). **F**, **G** Selected frames from time-lapse confocal movies of RFP-tagged EV and Rab37-WT RAW264.7 cells co-transfected with GFP-tagged ST2 cells. Enlarged images of the boxed areas with time intervals in seconds are shown (right). Arrow indicates trafficking vesicle (**F**). Quantitative analysis of Rab37 colocalization with ST2L over time (**G**). Vesicles of EV or Rab37-WT RAW264.7 cells expressing V5-tagged Rab37, were collected by centrifugations and immunoprecipitated (IP) with anti-V5 (**H**), and vesicle lysates were blotted for V5-Rab37 and endogenous ST2L (**I**). **J** Confocal microscopy images of Rab37 (red) and ST2L (green) in EV, Rab37-WT, Rab37-Q89L or Rab37-T43N RAW264.7 cells. **K** Flow cytometry analysis of ST2L membrane presentation in RAW264.7 cells overexpressing EV, Rab37-WT, Rab37-Q89L, and Rab37-T43N treated with rIL-33 (50 ng/mL). Scale bars, 10 μm. Data are presented as mean ± SD. *p < 0.05, **p < 0.01, ***p < 0.001, Student’s *t* test.

Next, we explored the key regulators in ST2L membrane trafficking in macrophages, focusing on the Rab family of small GTPase involved in vesicle trafficking. TCGA analysis revealed Rab37 with the strongest positive correlation with M2 macrophage infiltration in both LUAD and LUSC datasets (Fig. 2A, Supplementary Fig. S2A, B). Notably, *Rab37* mRNA expression significantly increased in rIL-13-induced M2 macrophages (Supplementary Fig. S2C). In addition, our previous study has revealed that Rab37 mediates the secretion of sST2 in lung cancer epithelial cells [15]. These observations prompted us to hypothesize Rab37 as a mediator of ST2L membrane presentation in M2 macrophages.

We generated WT and *Rab37* knockout (KO) C57BL/6 mice and isolated BMDMs. Using membrane fraction assay, we observed that *Rab37* KO BMDMs diminished ST2L membrane presentation compared to WT BMDMs (Fig. 2B). Flow cytometry analysis confirmed increased ST2L membrane presentation in WT BMDMs with M2 stimulus rIL-13 treatment (Fig. 2C). Of note, the combination of rIL-33 and rIL-13 further upregulated ST2L membrane presentation in WT BMDMs (Fig. 2C), but not in *Rab37* KO BMDMs (Fig. 2C). Furthermore, flow cytometry showed a significant increase of plasma membrane ST2L in the WT THP-1 overexpressing ST2L (ST2L OE), but not in *Rab37* KO THP-1 (Supplementary Fig. S2D). Together, these results suggested Rab37-mediated ST2L membrane presentation during IL-33/ST2L signaling transduction in macrophages.

### Rab37 mediates ST2L membrane presentation in macrophages in a GTP-dependent manner upon IL-33 stimulation

To clarify whether Rab37-mediated ST2L membrane presentation in macrophages depends on IL-33 stimulation, we conducted immunofluorescence (IF) confocal analysis on WT- and *Rab37* KO-BMDMs treated with rIL-33 (0–60 min). Confocal imaging revealed increased intracellular ST2L (green) at 15 min, with peak colocalization with Rab37 (red) at 30 min in WT-BMDMs, an IL-33-dependent process (Fig. 2D, E), not observed in *Rab37* KO-BMDMs (Supplementary Fig. S2E). Notably, an increased proportion of ST2L was located at the plasma membrane in WT-BMDMs after 60 min of rIL-33 treatment (Fig. 2D). To visualize the dynamic movements of ST2L trafficking regulated by Rab37, we performed time-lapse confocal microscopy on RAW264.7 macrophage cells co-transfected with GFP-ST2L vector and RFP-tagged empty vector (EV) or RFP-Rab37-WT vector. Imaging and quantitative results showed that ST2L was taken up into Rab37 punctate vesicular structures, forming colocalization vesicles (yellow), and subsequently released for another run of Rab37-mediated trafficking in Rab37-WT RAW264.7cells (Fig. 2F, G, Supplementary Movie S1, 2). Vesicle isolation analyses confirmed ST2L as a cargo protein of Rab37-specific vesicles (Fig. 2H), with elevated ST2L in Rab37-WT overexpressing RAW264.7 cells (Fig. 2I).

To assess if Rab37 mediates ST2L membrane presentation in macrophages in a GTP-dependent manner, we conducted confocal IF on RAW264.7 cells overexpressing EV, Rab37-WT, GTP-bound active Rab37-Q89L, and GDP-bound inactive Rab37-T43N. Results illustrated upregulated ST2L colocalizing with Rab37 in Rab37-WT and Rab37-Q89L cells compared to EV and Rab37-T43N groups (Fig. 2J). Notably, membrane fractionation and Western blotting analyses showed increased membrane-bound ST2L in Rab37-WT and Rab37-Q89L cells after rIL-33 treatment, compared to EV and Rab37-T43N groups (Supplementary Fig. S2F). Flow cytometry confirmed enhanced ST2L membrane presentation in Rab37-WT and Rab37-Q89L RAW264.7 cells, not in Rab37 T43N group (Fig. 2K). Altogether, these results suggested a positive-feedback mechanism where IL-33/ST2L signaling amplifies *ST2L* expression via NF-κB activation, followed by Rab37 mediating ST2L trafficking and membrane presentation in macrophages in a GTP-dependent manner, promoting M2 macrophage polarization.

### IL-33 or ST2L-neutralizing antibody treatment attenuates IL-33-mediated NF-κB activation and M2 macrophage polarization

**Fig. 3:**
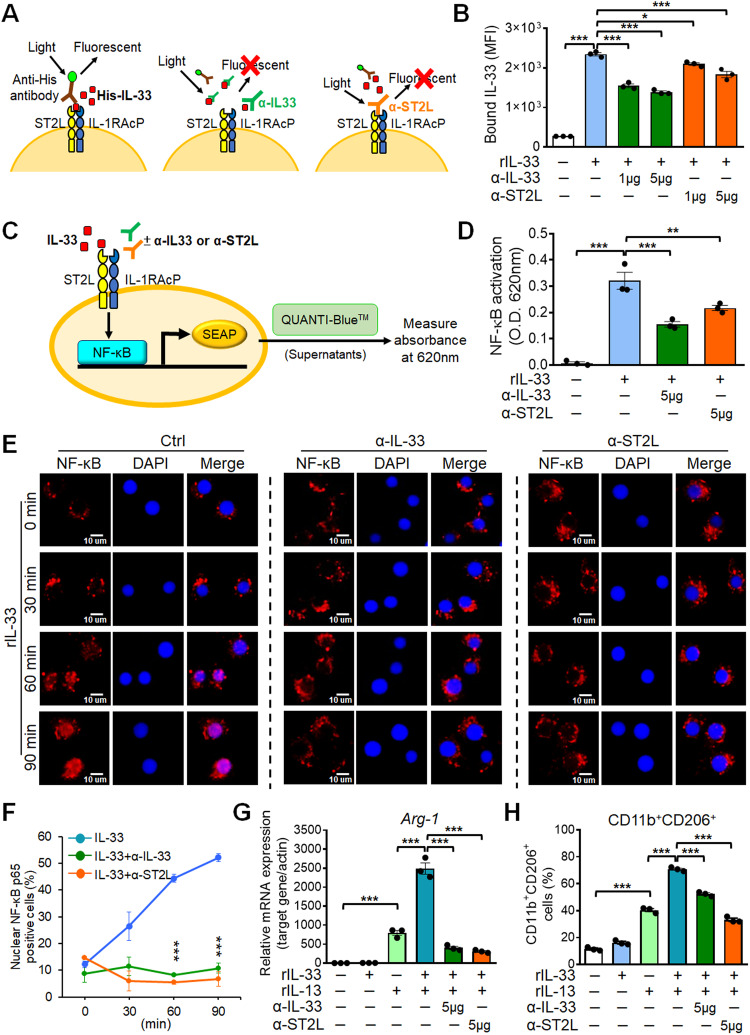
Neutralizing-IL-33 and -ST2L antibodies inhibit IL-33-mediated NF-κB activation and M2 macrophage polarization. **A** The scheme depicting His-IL-33 added to culture media of RAW264.7 cells emit fluorescence when bound to receptor (left). Neutralizing-IL-33 (α-IL-33) or -ST2L (α-ST2L) antibody attenuated fluorescence, indicating that α-IL-33 or α-ST2L antibodies blocked the interaction between His-IL-33 and receptor (middle and right). **B** Quantification of fluorescence signals in **A** from three independent experiments by flow cytometry. **C** Schematic diagram of NF-κB activity in RAW-Blue™ cells. Absorbance at 620 nm indicated the NF-κB activity. **D** α-IL-33 or α-ST2L Ab inhibited rIL-33-triggered NF-κB activation. **E**, **F** IF staining of NF-κB (red) nuclear translocation and DAPI (blue) in RAW264.7 cells treated with rIL-33 (at 0, 30, 60 and 90 min) and α-IL-33 or α-ST2L antibodies (**E**). The percentage of nuclear NF-κB-positive cells and quantification data are shown in the right (**F**). **G** RT-qPCR analysis of M2 markers *Arg-1* in RAW264.7 cells treated with rIL-13 (20 ng/mL) + rIL-33 (100 ng/mL) and α-IL-33 or α-ST2L antibodies. **H** Flow cytometry analysis of CD11b^+^CD206^+^ M2 macrophages population in RAW264.7 cells treated with rIL-13 (20 ng/mL) + rIL-33 (100 ng/mL) and α-IL-33 or α-ST2L antibodies. Scale bar, 10 μm. Data are presented as mean ± SD. *p < 0.05, **p < 0.01, ***p < 0.001, Student’s *t* test.

To abolish the pro-tumor effects of IL-33/ST2L signal in TME, we generated IL-33-neutralizing (α-IL-33) and ST2L-neutralizing antibody (α-ST2L) (Supplementary Fig. S3A, B) and examined their effects on macrophage polarization. First, we investigated whether α-IL-33 or α-ST2L blocked the binding of IL-33 to the ST2L receptor on RAW264.7 cells by flow cytometry assay. We used anti-His antibody to detect the His-tagged rIL-33 (His-IL-33) (*Left*, Fig. 3A), which showed increased IL-33 binding on RAW264.7 cells (bars blue *vs*. white, Fig. 3B). However, both α-IL-33 and α-ST2L (*Middle* and *Right*, Fig. 3A) decreased the fluorescence signals induced by rIL-33 (bars green and orange *vs*. blue, Fig. 3B), suggesting that α-IL-33 and α-ST2L prevented IL-33 binding to the ST2L receptor on RAW264.7 cells.

Next, we determined whether α-IL-33 or α-ST2L inhibited NF-κB activity in RAW-Blue^TM^ reporter cells with NF-κB-inducible secreted protein (Fig. 3C). The NF-κB reporter cell assay showed that α-IL-33 and α-ST2L inhibited rIL-33 binding to RAW-Blue™ cells and subsequently decreased the NF-κB activity upon rIL-33 treatment (Fig. 3D). Furthermore, Western blotting indicated that α-IL-33 and α-ST2L inhibited NF-κB phosphorylation (p-p65) induced by IL-33 in RAW264.7 cells (Supplementary Fig. S3C). In addition, IF staining revealed that IL-33 induced NF-κB nuclear translocation, which was reduced by α-IL-33 or α-ST2L treatment (Fig. 3E, F). Of note, RT-qPCR and flow cytometry showed that α-IL-33 and α-ST2L downregulated M2 markers *Arg-1* and *Ym-1* and reduced CD11b^+^ CD206^+^ M2 macrophages population compared to the rIL-13 and rIL-33 combination group (Fig. 3G, H and Supplementary Fig. S3D). Collectively, these data suggest that treatment of α-IL-33 and α-ST2L inhibits IL-33-mediated NF-κB activation and M2 polarization in macrophages.

### Cisplatin induces the release of IL-33 from lung cancer cells to promote M2 polarization

**Fig. 4:**
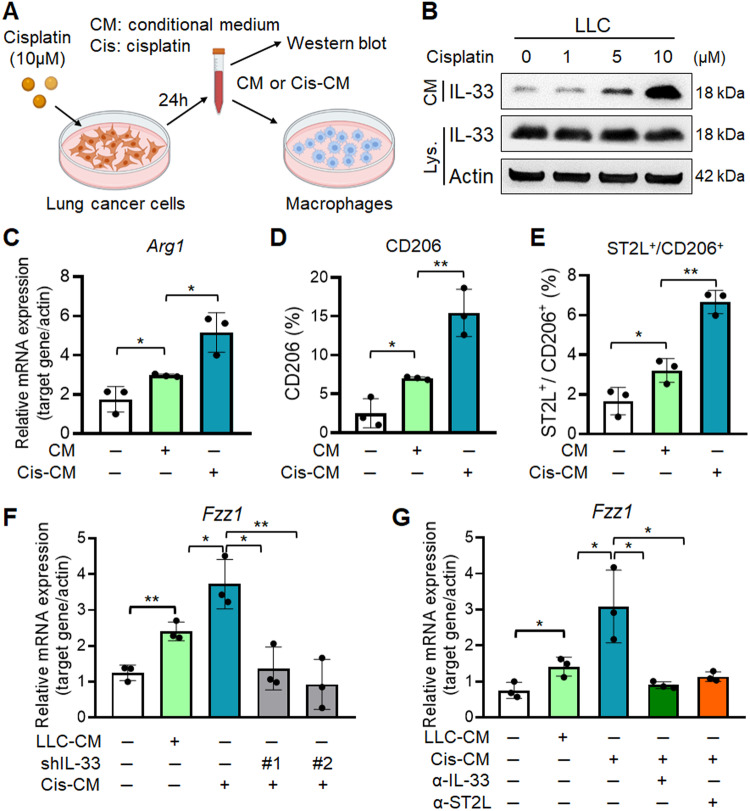
Cisplatin induces IL-33 secretion from cancer cells to promote M2 macrophage polarization. **A** Schematic diagram of RAW264.7 cells treated with the conditioned medium (CM) from LLC without or with cisplatin treatment (cis-CM). **B** Western blot analysis of IL-33 in the CM of LLC cells treated with 0, 1, 6 and 10 μM of cisplatin for 24 h. **C** RT-qPCR analysis of *Arg1* expression in RAW264.7 cells treated with CM or cis-CM from LLC cells for 24 h. Flow cytometry analysis of CD206^+^ (**D**) and ST2L^+^/CD206^+^ (**E**) populations in RAW264.7 cells treated with CM or cis-CM from LLC cells. **F** RT-qPCR analysis of *Fizz1* mRNA expression in RAW264.7 macrophages treated with Cis-CM from control LLC or IL-33 knockdown LLC cells. **G** RT-qPCR analysis of *Fizz1* mRNA expression in RAW264.7 cells treated with Cis-CM with or without treatment of α-IL-33 or α-ST2L antibodies. Data are presented as mean ± SD. *p < 0.05, **p < 0.01, ***p < 0.001, Student’s *t* test.

Previous studies observed that cisplatin treatment induces NRF2-mediated antioxidant response in tumor-initiating cells to trigger the extracellular release of the ‘danger’ signal IL-33 [7, 27]. We hypothesized that cisplatin-induced stress in LLC cancer cells causes IL-33 release, promoting M2 macrophage polarization. First, we explored whether cisplatin induced IL-33 release in LLC by conditional medium-western blot (CM-WB) (Fig. 4A). The results showed that the IL-33 levels in CM were increased in cisplatin-treated LLC or A549 in a dose-dependent manner (Fig. 4B and Supplementary Fig. S4A). Moreover, we examined whether the CM of untreated (CM) or cisplatin-treated lung cancer cells (Cis-CM) promoted M2 macrophage polarization in RAW264.7 and THP-1 cell lines (Fig. 4A). RT-qPCR and flow cytometry confirmed increased M2 marker *Arg1* mRNA and higher CD206^+^ and ST2L^+^/CD206^+^ M2 populations in macrophages treated with Cis-CM (Fig. 4C–E and Supplementary Fig. S4B).

We further performed the knockdown (KD) of IL-33 in LLC and CM-WB confirmed the reduction of secreted IL-33 upon cisplatin treatment (Supplementary Fig. S4C). The IL-33 KD in LLC significantly attenuated the Cis-CM-induced M2 macrophages (Fig. 4F), similar to α-IL-33 and α-ST2L treatments (Fig. 4G). These results suggested that cisplatin triggers M2 polarization by releasing of danger signal IL-33 from cancer cells.

### Blockade of IL-33 and ST2L enhances the anti-tumor effect of cisplatin by reducing cisplatin-induced immunosuppressive responses

Our in vitro data indicated that cisplatin promoted IL-33 release in cancer cells, while α-IL-33 and α-ST2L reversed the Cis-CM-induced M2 macrophages. Importantly, the cancer cell viability was significantly reduced by combination treatment consisting of α-IL-33 or α-ST2L and cisplatin (Supplementary Fig. S5A, B). Thus, we investigated the responses of α-IL-33 or α-ST2L in cisplatin-treated tumors in vivo.

**Fig. 5:**
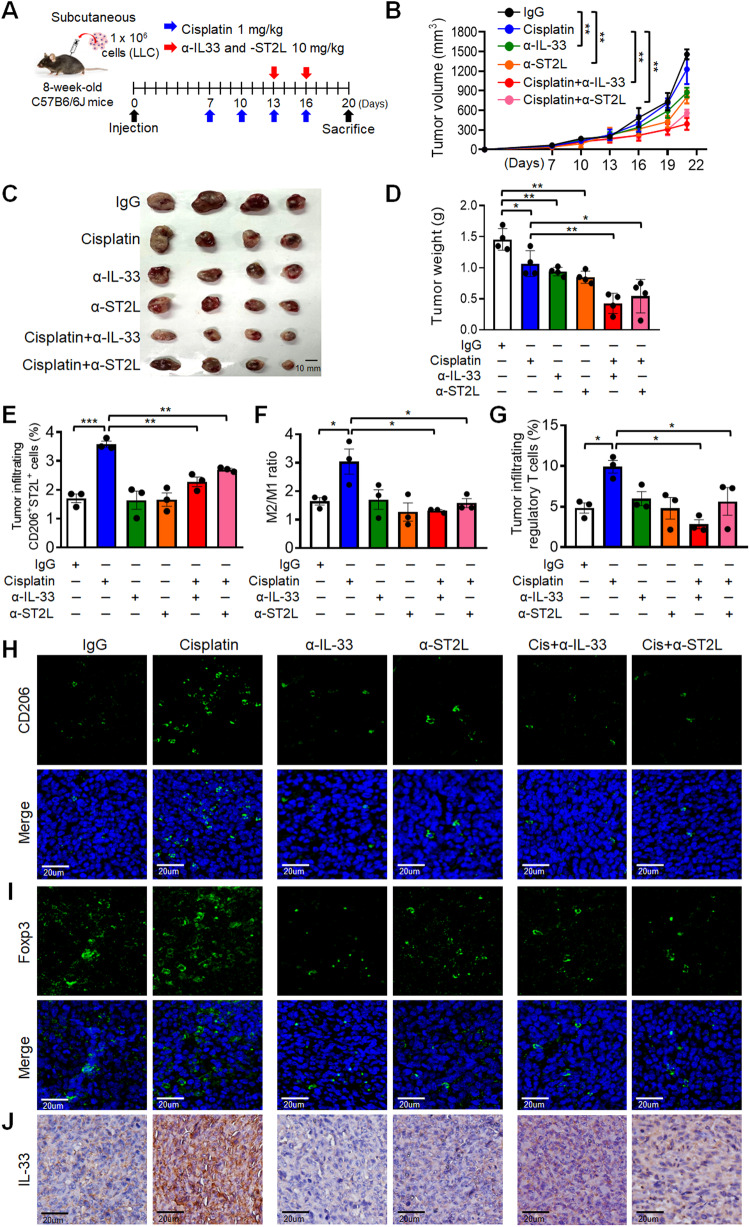
Combination of α-IL-33 or α-ST2L antibody enhances the anti-tumor effect of cisplatin in LLC allograft model. **A** Schematic representation of LLC allograft model and treatment strategy. **B**–**D** Tumor growth of LLC allografts treated with IgG, cisplatin, α-IL-33, α-ST2L, and cisplatin combined with α-IL-33 or α-ST2L (*n* = 4 per group). Tumor volume (**B**), tumor size (**C**) and tumor weight (**D**) were measured at the end of the experiment. Flow cytometric analysis of CD206^+^ ST2L^+^ M2 macrophages infiltration (**E**), M2 macrophages (CD206) to M1 (CD86) (M2/M1) ratio (**F**) and CD4^+^/CD25^+^/Foxp3^+^ regulatory T cells (Tregs) (**G**) in LLC tumors from each group. **H**, **I** Representative images of IF-immunohistochemistry (IF-IHC) staining of tumor sections from each group. Panels of CD206 macrophages (**H**) and Foxp3 Tregs (**I**) staining are shown as indicated. Nuclei were counterstained with DAPI (blue). **J** IL-33 IHC staining of tumor sections from each group. Scale bar, 20 μm. Data are presented as mean ± SD. *p < 0.05, **p < 0.01, ***p < 0.001, Student’s *t* test.

In the LLC allograft model, we assessed the anti-tumor effects of α-IL-33 or α-ST2L alone and in combination with cisplatin (Fig. 5A). While cisplatin alone partially reduced tumor growth, as indicated by volume, size, and weight, combining it with α-IL-33 or α-ST2L significantly enhanced tumor suppression (Fig. 5B–D). The serum biochemical markers showed no notable adverse effects from these treatments (Supplementary Fig. S5C–G).

In addition to ST2L-expressing macrophages, previous studies have confirmed that IL-33 induces the accumulation of Tregs to promote tumor growth and metastasis [8, 28]. Therefore, we further used flow cytometry to analyze immune cell infiltration in endpoint tumors, indicating that the percentage of CD206^+^ ST2L^+^ M2 macrophages, M2/M1 ratio and CD4^+^ Foxp3^+^ Tregs were higher in tumor allografts from the cisplatin monotherapy group (Fig. 5E–G). Notably, cisplatin-combined α-IL-33 or α-ST2L treatment groups reduced tumor-infiltrating M2 macrophages and Tregs compared to the cisplatin monotherapy group (Fig. 5E–G). The fluorescent immunohistochemistry (IF-IHC) staining also revealed that cisplatin-combined α-IL-33 or α-ST2L treatment suppressed the amount of tumor-infiltrating M2 macrophages and Tregs and reduced expression of PD-L1 immunosuppressive molecule, while increasing tumor-infiltrating CD8 T cells in LLC tumors (Fig. 5H, I and Supplementary Fig. S5H, I). Interestingly, IHC staining demonstrated that the combination of α-IL-33 or α-ST2L with cisplatin reversed cisplatin-induced IL-33 signal in tumor allografts (Fig. 5J). These results supported that blocking IL-33/ST2L signal enhanced cisplatin anti-tumor effect to inhibit tumor growth by reversing cisplatin-induced immunosuppressive TME.

### Rab37^+^ST2^+^CD206^+^ tumor-associated macrophages correlate with poor prognosis in NSCLC patients

**Fig. 6:**
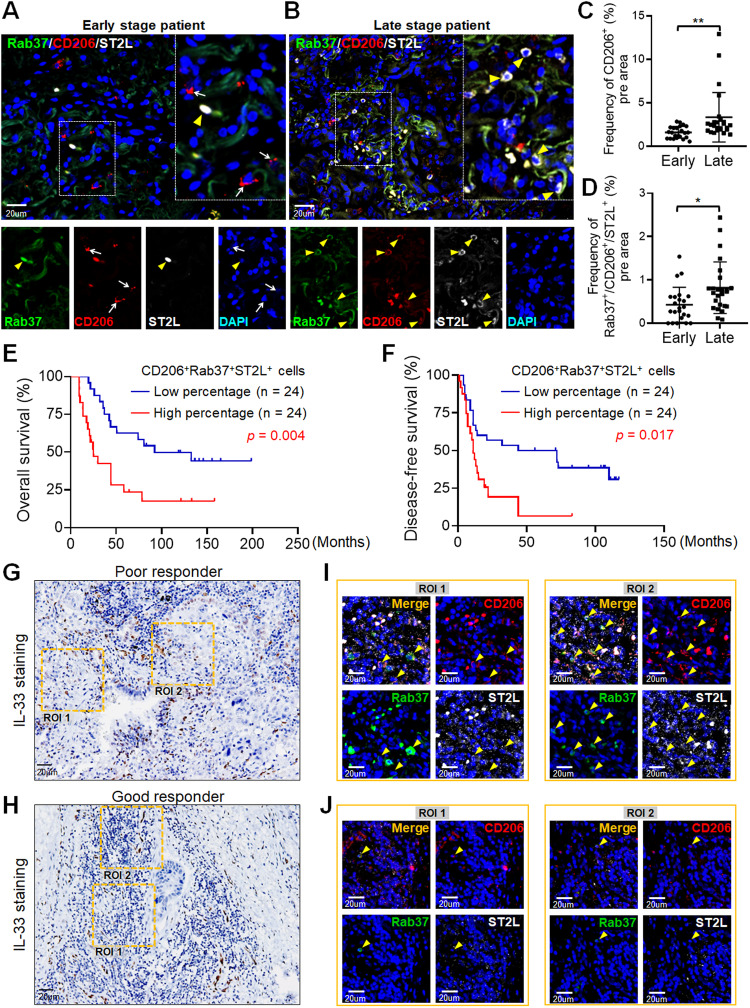
Lung cancer patients with high Rab37^+^ST2L^+^CD206^+^ expression correlate with poor survival and treatment response. Representative images of multi-color fluorescent IF-IHC staining of Rab37 (green), ST2L (red), and CD206 (white) in lung cancer tissues from early-stage (**A**) and late-stage (**B**) patients. Quantification of the percentage of CD206^+^ tumor-associated M2 macrophages (**C**) or the percentage of Rab37^+^ST2L^+^CD206^+^ tumor-associated M2 macrophages (**D**) indicated by three regions of interest (ROIs) for each tumor section in early- and late-stage lung cancer patients. Kaplan–Meier analysis of overall survival (**E**) and disease-free survival (**F**) of lung cancer patients with high and low tumor infiltrating Rab37^+^ST2L^+^CD206^+^ tumor-associated M2 macrophages. *p* values determined using log-rank test. Representative IHC images of IL-33 staining in lung cancer tissue sections from poor responder (**G**) and good responder (**H**) after chemotherapy. Enlarged IF-IHC images of the selected ROIs of Rab37 (green), CD206 (red), and ST2L (white) staining in lung cancer tissue sections from poor responder (**I**) and good responder (**J**). Scale bar, 20 μm. Data are presented as mean ± SD. *p < 0.05, **p < 0.01, ***p < 0.001, Student’s *t* test.

Thus far, our results indicated that IL-33 promotes M2 macrophage polarization and an immunosuppressive TME, reducing cisplatin efficacy in lung cancer via IL-33/ST2L/NF-κB signaling and Rab37/ST2L trafficking. To verify whether Rab37^+^ST2L^+^CD206^+^ tumors elicited an immunosuppressive TME, we performed multi-color IF-IHC to detect Rab37, ST2L, and CD206 in surgical tumor specimens of 48 lung cancer patients. Figure 6A, B illustrates one tumor from the early-stage patient and another from the advanced-stage patient. In early-stage patients, ST2L and Rab37 showed low colocalization in CD206-labeled tumor-associated M2 macrophages (Fig. 6A), contrasting with increased colocalization in advanced lung cancer (Fig. 6B). Next, we quantitatively analyzed the percentage of CD206^+^ tumor-associated M2 macrophages on multiple regions of interest (ROIs), normalized to nuclei staining for each patient. The results showed a higher frequency of CD206^+^ tumor-associated M2 macrophages in late-stage lung cancer patients than in early-state patients (Fig. 6C). Furthermore, the correlation between Rab37^+^ST2L^+^CD206^+^ tumor-associated M2 macrophages was higher in advanced-stage patients (Fig. 6D). These clinical results suggest that Rab37-mediated ST2 presentation is important in tumor-associated M2 macrophages during tumor progression.

### Rab37^+^ST2^+^CD206^+^ tumor-associated macrophages correlate with poor response to chemotherapy in NSCLC patients

Moreover, we conducted correlation analysis, relating intratumoral Rab37^+^ST2L^+^CD206^+^ tumor-associated M2 macrophage stain intensity to clinicopathological parameters of 48 lung cancer patients. High tumor-infiltrating Rab37^+^ST2L^+^CD206^+^ signals correlated with advanced tumor stages and cancer recurrence (Table 1). Additionally, univariate Cox regression indicated that lung cancer patients with high tumor-infiltrating Rab37^+^ST2L^+^CD206^+^ tumor-associated M2 macrophage profile had increased death risk. Importantly, multivariate Cox regression analysis (hazard ratio = 2.311, *p* = 0.028) confirmed this risk, even adjusting for clinical parameters (Table 2). The Kaplan–Meier analysis revealed that higher infiltrating Rab37^+^ST2L^+^CD206^+^ tumor-associated M2 macrophages correlated with poorer overall and disease-free survival in lung cancer patients (Fig. 6E, F).

**Table 1:** Alteration of CD206^+^Rab37^+^ST2L^+^ cells in relation to clinicopathological parameters in 48 lung cancer patients’ tumor specimens.

Clinical		Total	CD206^+^Rab37^+^ST2L^+^ cells (%)		
Parameters		Patients	Protein Expression		*p*-value^a^
		48	*N* = 24	*N* = 24	
			(50%)	(50%)	
			Low expression^b^	High expression^b^	
Age	<65	34	15 (44.1)	19 (55.9)	0.171
	≥65	14	9 (64.3)	5 (35.7)	
Gender	Male	26	15 (57.7)	11 (42.3)	0.193
	Female	22	9 (40.9)	13 (59.1)	
Tumor stage	I-II	23	15 (65.2)	8 (34.8)	0.041
	III-IV	25	9 (36)	16 (64)	
T stage^c^	T1-2	38	21 (55.3)	17 (44.7)	0.143
	T3-4	10	3 (30.0)	7 (70.0)	
N stage^c^	≤N1	14	11 (78.6)	3 (21.4)	0.012
	>N1	34	13 (38.2)	21 (61.8)	
M stage^c^	M0	42	23 (54.8)	19 (45.2)	0.094
	≥M1	6	1 (16.7)	5 (83.3)	
Differentiation grade	Well	8	4 (50.0)	4 (50.0)	0.287
	Moderate	32	18 (87.5)	14 (12.5)	
	Poor	8	2 (25)	6 (75)	
Recurrence	No	13	12 (92.3)	1 (7.7)	**0.001**
	Yes	35	12 (34.3)	23 (65.7)	

^a^The data were analyzed by Pearson χ^2^ test. *p-*values with significance are shown as bold letters.

^b^Patient with CD206^+^Rab37^+^ST2L^+^cells (%) ≥ 0.6% of ROIs were defined as high expression.

^c^T stage: primary tumor; N stage: lymph node metastasis; M stage: distant metastasis.

**Table 2:** Cox regression analysis of risk factors for cancer-related death in lung cancer patients.

Characteristics		Univariate analysis		Multivariate analysis	
		HR^a^ (95% CI^b^)	*p*-value^c^	HR (95% CI)	*p*-value^c^
CD206^+^Rab37^+^ST2L^+^ cells (%)^d^	<0.6%	1.00		1.00	
	≥0.6%	2.824(1.375–5.801)	**0.005**	2.311 (1.096–4.875)	**0.028**
Age	<65	1.00		–^f^	
	≥65	0.437 (0.187–1.019)	0.055	–^f^	–^f^
Gender	Female	1.00		–^f^	
	Male	1.090 (0.544–2.184)	0.809	–^f^	–^f^
Tumor stage	I-II	1.00		1	
	III-IV	2.636 (1.251–5.555)	0.011	2.103 (0.971–4.557)	0.060
T status^e^	T1-2	1.00		–^f^	
	T3-4	1.680 (0.744–3.791)	0.212	–^f^	–^f^
N status^e^	≤N1	1.00		–^f^	
	>N1	1.753 (0.784–3.920)	0.171	–^f^	–^f^
M status^e^	M0	1.00		1.00	
	≥M1	2.721 (1.084–6.830)	**0.033**	1.450 (0.542–3.875)	0.459

^a^*HR* Hazard ratio.

^b^*CI* Confidence interval.

^c^Bold values indicate statistical significance (*p* < 0.05).

^d^Patient with CD206^+^Rab37^+^ST2L^+^cells (%) ≥ 0.6% of ROIs was defined as high expression.

^e^T status: primary tumor; N status: lymph node metastasis; M status: distant metastasis.

^f^Variables without significant HR in the univariate analysis were not included in the multivariate analysis.

Finally, we assessed IL-33 expression and Rab37^+^ST2L^+^CD206^+^ M2 macrophage infiltration in lung cancer patients with chemotherapy response data. Poor responders showed elevated IL-33 levels (Fig. 6G, H) and increased infiltrating Rab37^+^ST2L^+^CD206^+^ tumor-associated M2 macrophages (yellow arrow) (Fig. 6I, J). These clinical data suggest that tumors from lung cancer patients with poor survival rates or poor chemo-responses are characterized by a distinct immunosuppressive TME with a high level of infiltrating Rab37^+^ST2L^+^CD206^+^ tumor-associated M2 macrophages. A high level of tumor-infiltrating Rab37^+^ST2L^+^CD206^+^ M2 macrophages independently predicted clinical outcomes in lung cancer patients.

## Discussion

**Fig. 7:**
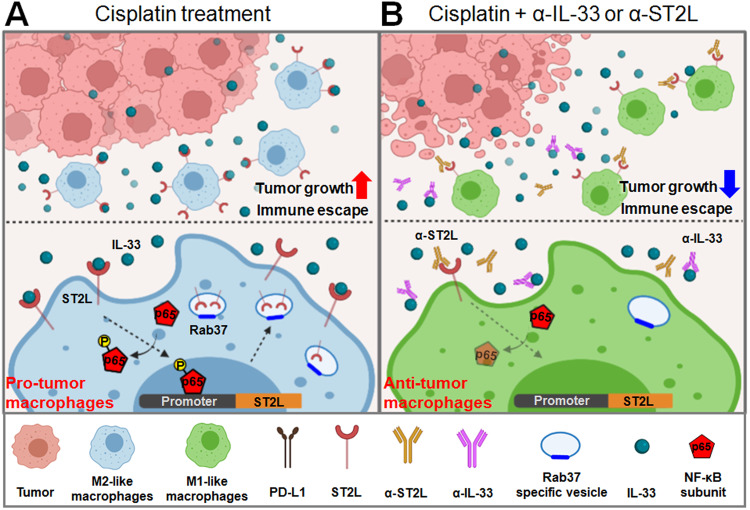
A schematic diagram showing that the IL-33/NF-κB/ST2L/Rab37 positive-feedback loop promotes M2 macrophage to limit chemotherapeutic efficacy in lung cancer. **A** The IL-33/NF-κB/ST2L/Rab37 pathway creates a positive feedback loop, leading to increased expression and membrane presentation of ST2L in M2 macrophages, enabling them to respond to the intratumor IL-33. **B** Blocking IL-33 or ST2L with specific antibodies can inhibit NF-κB activity, suppress the polarization of M2 macrophages, and significantly reduce tumor growth when combined with cisplatin treatment.

Our findings herein reveal a novel positive-feedback mechanism by which IL-33/ST2L signaling amplifies *ST2L* expression by NF-κB activation, followed by Rab37 mediating ST2L membrane trafficking on macrophages, leading to M2 macrophage polarization. Moreover, we demonstrated that cisplatin-treated lung cancer cells significantly increase IL-33 release, promoting M2 macrophage polarization and enhancing tumor cell immune escape. Notably, α-IL-33 or α-ST2L reversed the polarization of macrophages and enhanced the anticancer effect of cisplatin treatment. Together, our discovery of IL-33-induced ST2L transcription followed by Rab37-mediated ST2L trafficking in M2 macrophages, highlights the IL-33/ST2L axis as a promising target for combined chemotherapy and immunotherapy (Fig. 7).

Our in vitro results discovered that IL-33 drives a positive-feedback loop, enhancing M2 polarization by upregulating its receptor ST2L expression. Despite ChIP and promoter activity assays revealing IL-33-induced NF-κB activation in promoting *ST2L* transcription in M2 macrophages, additional transcription factors cannot be ruled out. Of note, we observed that treatment of JNK inhibitor reduced *ST2L* mRNA expression. Interestingly, the activator protein-1 (AP-1), a downstream transcription factor of JNK, was predicted to bind on −352~−360 of the *ST2L* promoter adjacent to site 2 of NF-κB (−360~−349) using PROMO software. Moreover, GATA-3 enhances *ST2L* gene expression by binding to an enhancer located 12 kb upstream of its transcription start site, whereas STAT5 binds to the *ST2L* promoter region [29, 30]. Therefore, investigating whether NF-κB cooperates with AP-1 and other transcription factors at promoter or enhancer for increased *ST2L* transcriptional in M2 macrophages is warranted.

IL-33/ST2L signaling regulates the functions of immune cells such as T cells, dendritic cells, macrophages, and innate lymphoid cells (ILCs) [31–34]. However, the membrane presentation mechanism of critical receptor ST2L is still unknown. Here, we provide a novel mechanism of Rab37-mediated ST2L membrane trafficking in a GTP-dependent manner in macrophages. Rab37 functions as a regulator of intracellular vesicle transport. Notably, Rab37 has been shown to promote the formation of the ATG5-12-16L1 complex in the initial autophagosomal formation [35]. We recently reported that Rab37 is involved in secretory autophagy to mediate TIMP1 secretion in lung cancer cells [36]. Moreover, Rab11 and Rab27, family members of Rab37, are pivotal in regulating exosome secretion [37]. Therefore, further exploration may unveil additional Rab proteins and mechanisms linked to ST2L membrane trafficking.

The IL-33/ST2 signaling pathway influences tumorigenesis in various cancers [8, 38]. Conversely, the IL-33/ST2L axis modulates the TME, recruiting immune cells and improving anti-tumor immunity [39–41]. The pleiotropic role of IL-33 in tumor development may depend on the stage of the tumor (early *vs*. late stages) and IL-33 status (acute *vs*. chronic release). Notably, the in vitro anti-tumor results indicated that the α-IL-33 and α-ST2L did not directly inhibit cell growth or induce cell death in LLC (Fig. 3E-H) presumably due to low basal level of IL-33 secretion in lung cancer cell lines without cisplatin treatment (Fig. 4B and Fig. 4A). Notably, in our in vivo study, the administered dose of cisplatin was 1 mg/kg, which was lower than the doses utilized in prior studies [42, 43]. Our in vivo approach aimed to achieve two main objectives: firstly, to enhance the therapeutic effect against tumors by leveraging the synergy between the antibodies and cisplatin, and secondly, to reduce the dosage of chemotherapeutic drugs to mitigate their side effects.

Clinical trials employing IL-33/ST2L neutralizing antibodies primarily target inflammatory diseases and immune disorders [5, 44–47]. Emerging preclinical studies are investigating IL-33/ST2L axis blockade to enhance anti-tumor immune response and overcome chemoresistance. For example, treatment of α-IL-33 and α-ST2L suppresses type 2 ILCs, inhibiting melanoma progression and reversing chemoresistance [48, 49]. Moreover, α-IL-33 increases tumor-specific CD8^+^ T cell infiltration in melanoma. Combining α-PD1 treatment synergistically enhanced the α-IL33 efficacy on tumor growth [48]. Our study showed that α-IL-33 and α-ST2L blocked the IL-33/ST2L axis and suppressed M2 macrophage polarization to enhance anti-tumor effect of cisplatin in lung cancer. Furthermore, α-IL-33 and α-ST2L treatments increased CD8^+^ T cell infiltration in the tumor site, which suggests that combination with α-PD-1 may enhance the anti-IL33 therapeutic efficacy in lung cancer.

In summary, we have discovered the new mechanism of a positive-feedback loop involving IL-33/NF-κB/ST2L transcription and Rab37/ST2L membrane trafficking in IL-33-induced M2 macrophage. Our pre-clinical evidence supports targeting pro-tumor cytokine IL-33 and its receptor ST2L as an effective strategy to convert the immune-evasive TME to immunoreactive TME for tumor therapy.

### Supplementary information

Supplementary Movie 1. Time-lapse movie of confocal images in empty vector (EV) RAW264.7 cells expressing GFP-tagged ST2L and RFP-tagged EV.

Supplementary Movie 2. Time-lapse movie of confocal images in Rab37 wild-type RAW264.7 cells expressing GFP-tagged ST2L and RFP-tagged Rab37.

Supplementary information

Uncropped Western Blots

## Data Availability

All data generated during this study are included in this published article and its supplementary information files.
