# Correction: IL-33/NF-κB/ST2L/Rab37 positive-feedback loop promotes M2 macrophage to limit chemotherapeutic efficacy in lung cancer

**DOI:** 10.1038/s41419-024-06879-0

**Published:** 2024-08-27

**Authors:** You-En Yang, Meng-Hsuan Hu, Yen-Chen Zeng, Yau-Lin Tseng, Ying-Yuan Chen, Wu-Chou Su, Chih-Peng Chang, Yi-Ching Wang

**Affiliations:** 1 Institute of Basic Medical Sciences, College of Medicine, National Cheng Kung University, No.1, University Road, Tainan, 701 Taiwan; 2 Department of Pharmacology, College of Medicine, National Cheng Kung University, No.1, University Road, Tainan, 701 Taiwan; 3 Department of Microbiology and Immunology, College of Medicine, National Cheng Kung University, No.1, University Road, Tainan, 701 Taiwan; 4 Division of Thoracic Surgery, Department of Surgery, College of Medicine, National Cheng Kung University, No.1, University Road, Tainan, 701 Taiwan; 5 Division of Oncology, Department of Internal Medicine, College of Medicine, National Cheng Kung University, No.1, University Road, Tainan, 701 Taiwan

**Keywords:** Mechanisms of disease, Cancer microenvironment, Transcription, Small GTPases, Cancer therapy

Correction to: *Cell Death and Disease* 10.1038/s41419-024-06746-y, published online 22 May 2024

In this Research Article, the first name of Ying-Yuan Chen was misspelled as ‘Ying-Yung’.

The original article has been corrected.

