# Supplementary material for: IL-33/NF-κB/ST2L/Rab37 positive-feedback loop promotes M2 macrophage to limit chemotherapeutic efficacy in lung cancer
Source: Cell Death Dis. 2024 May 22;15(5):356. doi: 10.1038/s41419-024-06746-y (PMC11111460; doi:10.1038/s41419-024-06746-y)
Supplement: Supplementary file 3 — Supplementary information [file 41419_2024_6746_MOESM3_ESM.pdf]

IL-33/NF-κB/ST2L/Rab37 positive-feedback loop promotes M2 macrophage to limit chemotherapeutic efficacy in lung cancer

Supplementary Figures and Tables

Supplementary materials and methods

Supplementary Fig. 1 is related to Fig. 1

Supplementary Fig. 2 is related to Fig. 2

Supplementary Fig. 3 is related to Fig. 3

Supplementary Fig. 4 is related to Fig. 4

Supplementary Fig. 5 is related to Fig. 5

Supplementary Movie 1 is related to Fig. 1

Supplementary Movie 2 is related to Fig. 1

Supplementary Table 1 is related to Materials and Methods.

Supplementary Table 2 is related to Materials and Methods.

Supplementary Table 3 is related to Materials and Methods.

Supplementary Movie 1

Supplementary Movie 2

Supplementary materials and methods

Quantitative reverse transcriptase-polymerase chain reaction (RT-qPCR) assay

Total RNA was extracted using Trizol reagent (Invitrogen). Purified RNA was converted into cDNA by reverse transcription. q-PCR was performed to analyze the mRNA expression of genes by using SYBR Green Master Mix (Invitrogen).

Protein extraction and Western blot analysis

The cells were harvested and lysed in RIPA buffer containing protease inhibitors cocktail (Sigma-Aldrich, St. Louis, MO, USA), and then cell lysates were centrifuged at 13,200 r.p.m. for 15 min at 4°C. Protein extracts were solubilized in loading buffer (60 mM Tris-base, 2% SDS, 10% glycerol, and 5% β-mercaptoethanol). A total of 50 μg of protein lysates was loaded onto 10% sodium dodecyl sulfate/ polyacrylamide gel electrophoresis (SDS-PAGE) and transferred onto a polyvinyl difluoride (PVDF) membrane. The protein was identified by incubating the PVDF membrane with primary antibodies followed by horse radish peroxidase-conjugated secondary antibodies.

Real-time live confocal fluorescence microscopy

V5-His tagged Rab37 wild-type (homemade) and pLV-mIL1RL1-GFPSpark (ST2L expression vector, Sino Biological Inc.) were transfected and expressed for 24 h in RAW264.7 cells. The Rab37-RFP and ST2L-GFP signals in RAW264.7 cells were recorded in real-time live images and video before and after the IL-33 50 *n*g/mL treatment were captured through the 100x lens of the microscope. The time-lapse images were acquired using an Olympus FV3000 confocal microscope with FV31S-SW software (Olympus, Tokyo, Japan).

Cell viability and cell death assay

To evaluate the cytotoxicity of cisplatin, α-IL-33 and α-ST2L antibodies (Leadgene Biomedical), cell viability and death assay were performed by cell counting kit 8 (CCK-8) assay (Dojindo Laboratories, Kumamoto, Japan) and lactate dehydrogenase (LDH) releasing assay (Roche, Basel, Switzerland). After cisplatin (10 μM), α-IL-33 (5 μg/mL) or α-ST2L (5 μg/mL) antibody treatment, cell culture media were replaced with fresh media containing CCK-8 reagent or LDH assay reagent and then incubated at 37°C for 30 min. For the CCK-8 assay, the optical absorbance at 450 nm was measured. For the LDH assay, the optical absorbance at 490 nm was measured.

Supplementary Figures and Legends

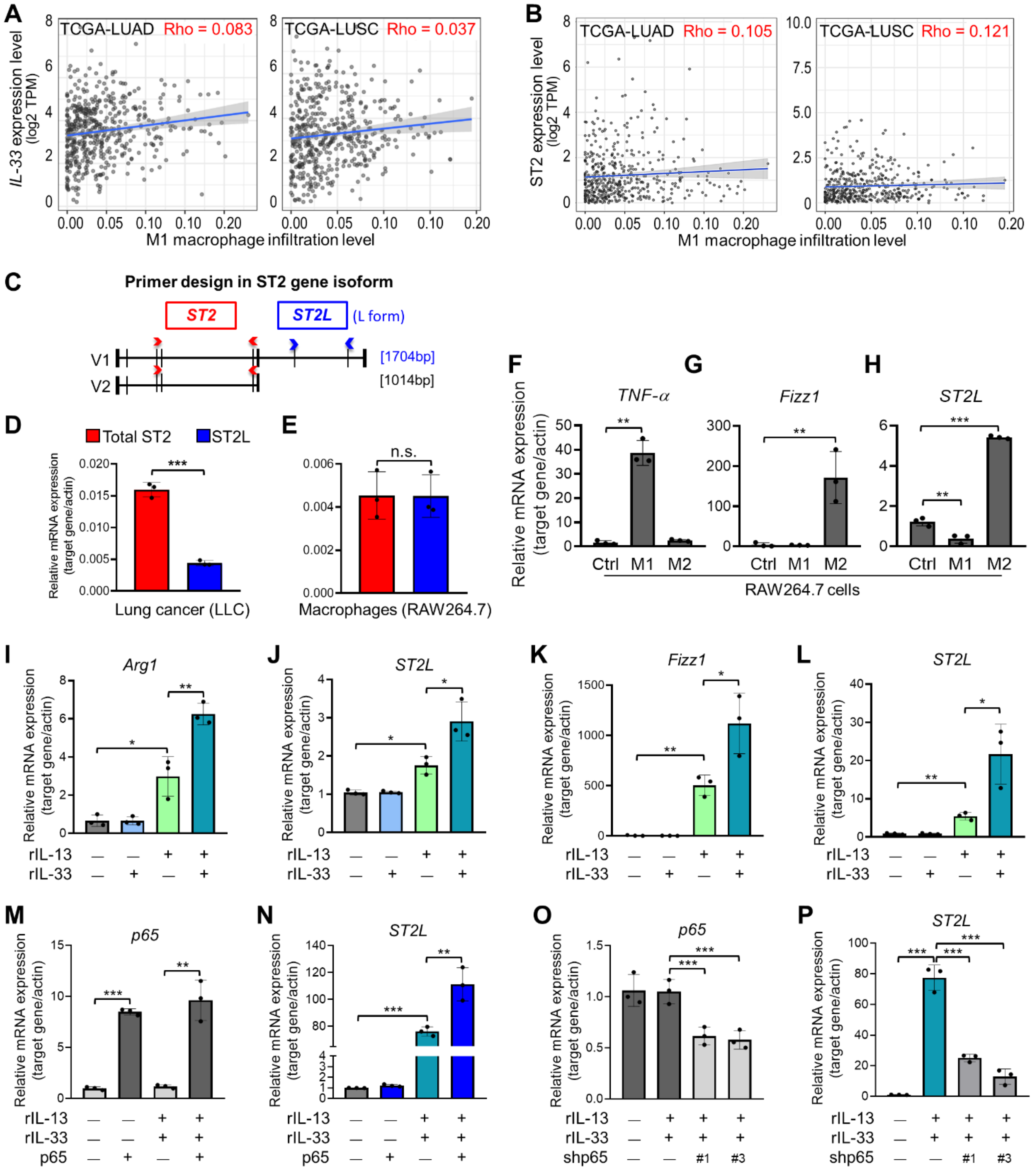

Supplementary Fig. 1 IL-33 and ST2L are expressed in macrophages and can be further induced in M2 macrophages. A, B Correlation analysis of *IL-33* mRNA expression level (**A**) or *ST2* mRNA expression level (**B**) with M1 macrophage infiltration in LUAD (n = 515) and LUSC (n = 501) using the TIMER2.0 database. **C** Primers designed for *ST2L* (V1) and the alternative splicing of *ST2* (V2). **D, E** RT-qPCR analyses were performed to examine the mRNA expression of *ST2* and *ST2L* transcripts in LLC cancer cells (**D**) and RAW264.7 macrophage cells (**E**). **F-H** mRNA expression analysis of M1 marker gene *TNF-α* (**F**), M2 marker gene *Fizz1* (**G**), and *ST2L* (**H**) in RAW264.7 cells treated with M1 stimuli (LPS 100 *ng*/mL combined IFNγ 100 IU for 24 h) or M2 stimuli (IL-13 20 *ng*/mL for 48 h). **I, J** RT-qPCR analysis of M2 marker gene *Arg1* (**I**) and *ST2L* (**J**) expression in RAW264.7 cells treated with rIL-13, M2 inducer alone (20 *ng*/mL), rIL-33 alone (100 *ng*/mL), or rIL-13 combined with rIL-33 for 48 h. **K, L** RT-qPCR analysis of M2 marker gene *Fizz1* (**K**) and *ST2L* (**L**) expression in BMDM cells treated with rIL-13 alone, rIL-33 alone,

or rIL-13 combined with rIL-33 for 48 h. **M, N** RT-qPCR analyses of *p65* (**M**) and *ST2L* (**N**) mRNA expression in THP-1 macrophage cells with p65 overexpression, following treatment with rIL-13 and rIL-33 for 48 h. **O, P** RT-qPCR analysis of *p65* (**O**) and *ST2L* (**P**) mRNA expression in THP-1 macrophage cells with p65 knockdown treated with rIL-13 and rIL-33 for 48 h. Data are presented as mean ± SD. *$p < 0.05$, **$p < 0.01$, ***$p < 0.001$, Student's *t*-test.

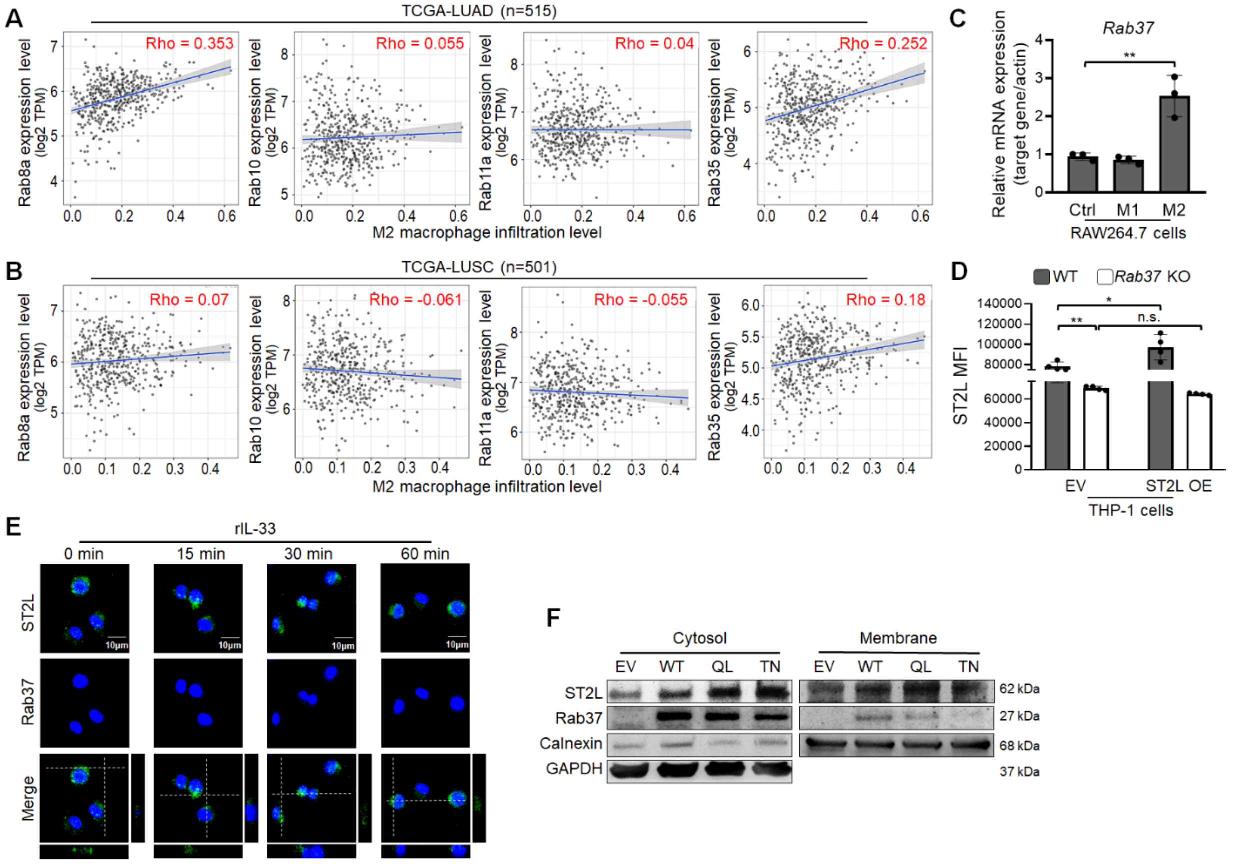

Supplementary Fig. 2 IL-33 enhances M2 polarization and ST2L expression in RAW264.7 cells. A, B Correlation analysis of mRNA expression level of Rab family members in LUAD (n = 515) (**A**) and LUSC (n = 501) (**B**) using the TIMER2.0 database. **C** mRNA expression of *Rab37* in RAW264.7 cells with M1 and M2 treatment. **D** Analyzing ST2L overexpression in WT and *Rab37* KO THP-1 cells using flow cytometry. **E** Confocal IF analysis of the localization of Rab37 (red) and ST2L (green) in *Rab37 KO* BMDMs treated with rIL-33 (50 ng/mL) at 0, 15, 30, and 60 min. **F** Western blotting analysis of membrane fraction proteins from RAW264.7 cells overexpressing EV, Rab37-WT, Rab37-Q89L, and Rab37-T43N treated with rIL-33 (50 *n*g/mL). Data are presented as mean ± SD. **p* < 0.05, ***p* < 0.01, ****p* < 0.001, Student's *t*-test.

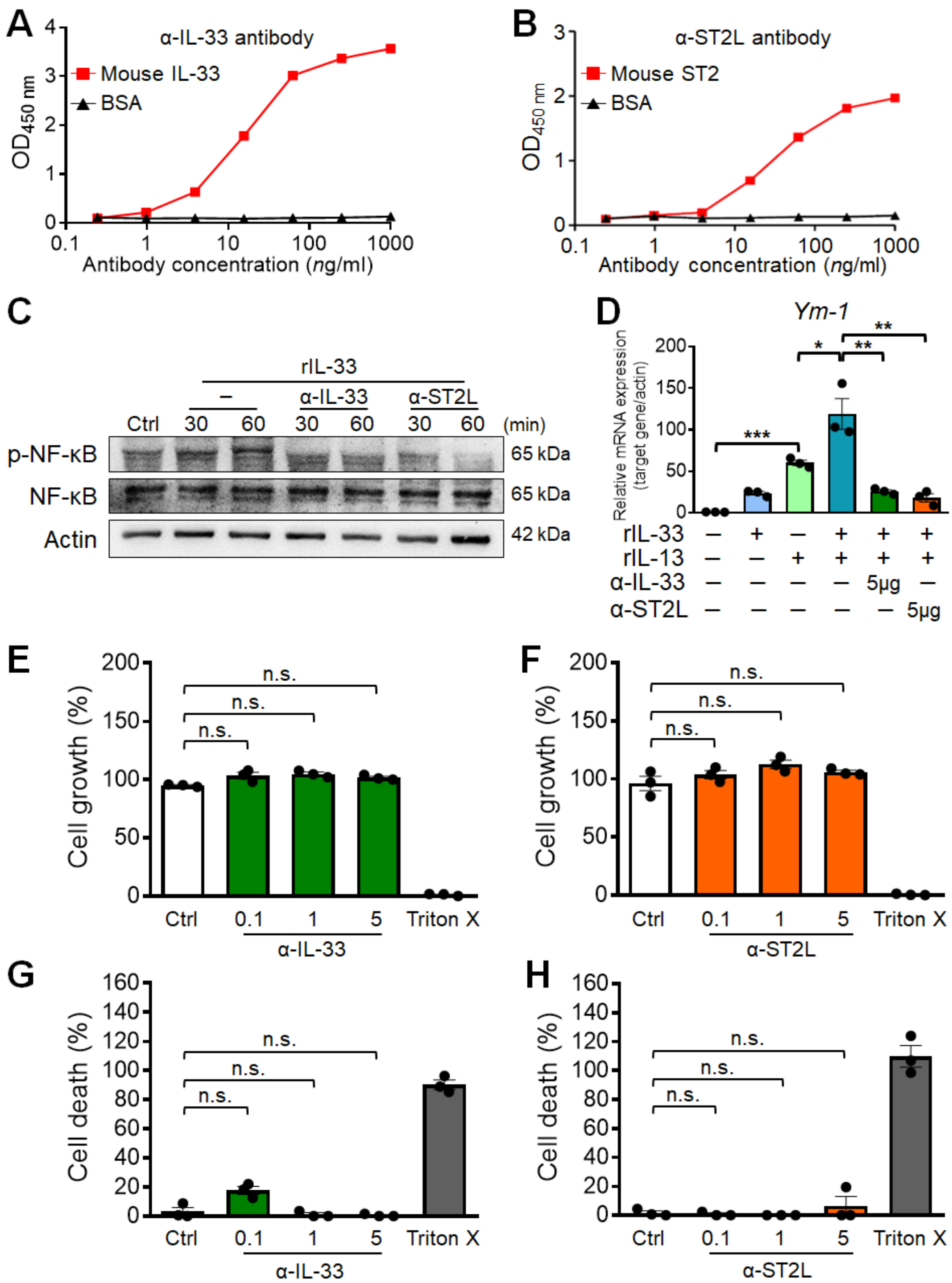

Supplementary Fig. 3 Neutralizing-IL-33 (α-IL-33) and -ST2L (α-ST2L) antibodies inhibit NF-κB phosphorylation induced by IL-33 in RAW264.7 cells, but do not directly affect LLC cell growth or induce cell death. A, B ELISA assay measuring the binding capacity of α-IL-33 antibody to mouse rIL-33 (**A**) or the binding capacity of α-ST2L antibody to mouse ST2 (**B**). **C** Western blotting analysis of NF-κB phosphorylation in RAW264.7 cells treated with IL-33 and α-IL-33 or α-ST2L antibodies. **D** RT-qPCR analysis of M2 markers *Ym-1* in RAW264.7 cells treated with rIL-13 (20 *ng*/mL) + rIL-33 (100 *ng*/mL) and α-IL-33 or α-ST2L antibodies. **E, F** Cell growth of LLC cells treated with α-IL-33 (**E**) or α-ST2L (**F**) antibodies at 0.1, 1, 5 μg/mL for 24 h. **G, H** Cell death of LLC cells treated with α-IL-33 (**G**) or α-ST2L (**H**) antibodies at 0.1, 1, 5 μg/mL for 24 h. Triton X was included as a positive control. Data are presented as mean ± SD. *$p < 0.05$, **$p < 0.01$, ***$p < 0.001$, n.s.: not significant. Student's *t*-test.

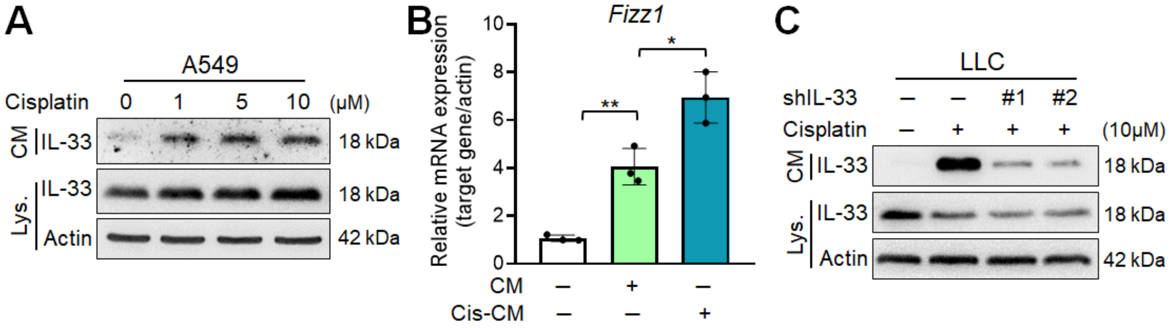

Supplementary Fig. 4 IL-33 released from cisplatin-treated lung cancer cells promotes M2 macrophage polarization. **A** Western blot to determine IL-33 levels in conditional media (CM) from A549 cells treated with cisplatin. **B** RT-qPCR analysis of *Fizz1* mRNA expression in THP-1 cells treated with Cis-CM (CM from cisplatin-treated A549) or control CM. **C** Knockdown efficiency of IL-33 in LLC cells was confirmed by Western blotting. Data are presented as mean ± SD. *$p < 0.05$, **$p < 0.01$, ***$p < 0.001$, Student's *t*-test.

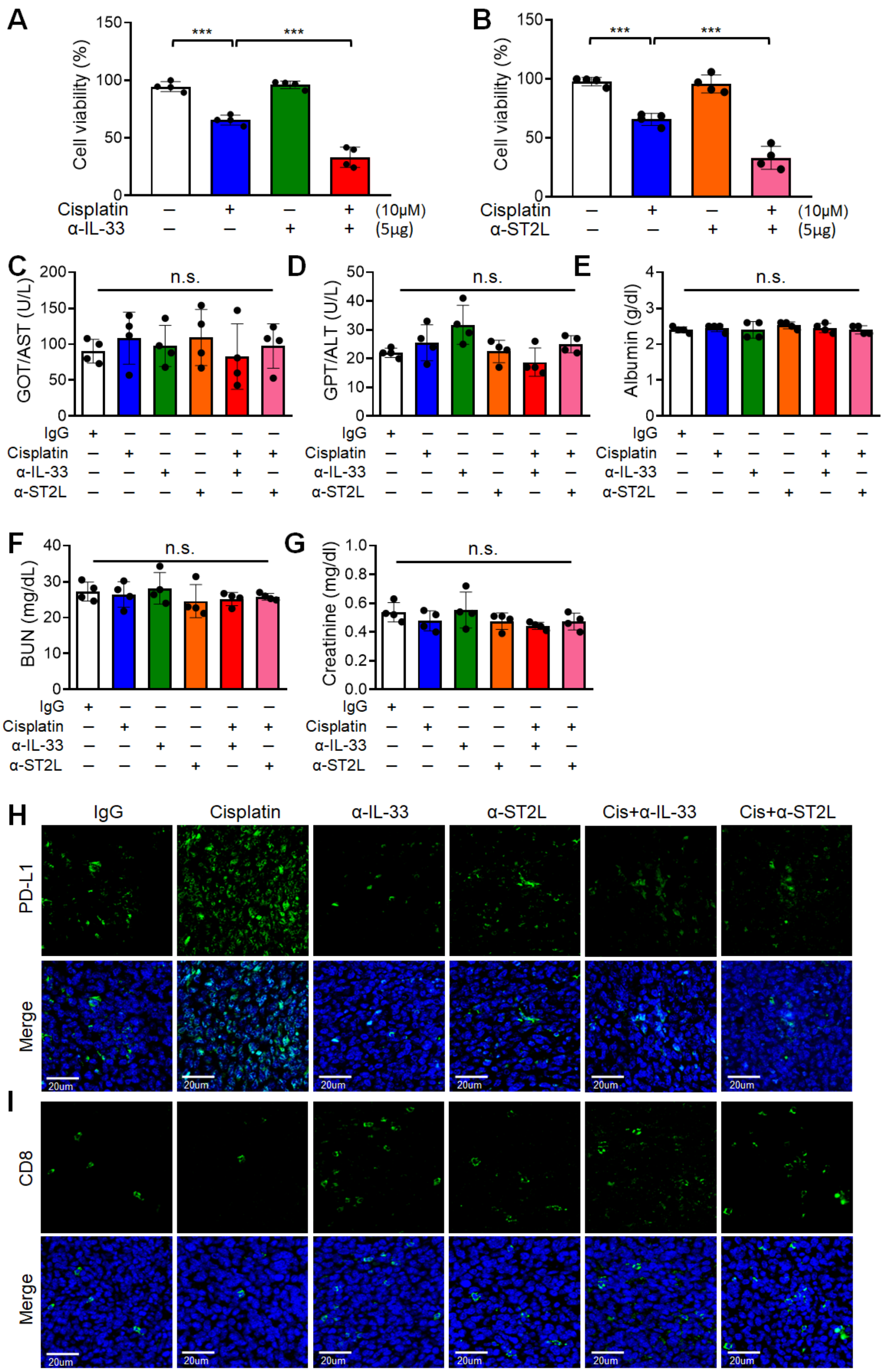

Supplementary Fig. 5 Effects of combination treatment with α-IL-33 or α-ST2L antibody and cisplatin on LLC cell viability, serum biochemical markers, and tumor immune microenvironment. A, B LLC cell viability after treatment with cisplatin (10 μM), α-IL-33 (5 μg/mL) (**A**) or α-ST2L (5 μg/mL) (**B**) antibody, or their combination. **C-G** Serum levels of biochemical markers (GOT, GPT, Albumin, BUN, and Creatinine) in treated mice from each group. **H, I** Representative images of IF-IHC staining of tumor sections showing PD-L1 (**H**) expression and infiltrated CD8 T cells (**I**) in treated mice from each group. Scale bar, 20 μm. Data are presented as mean ± SD. *$p < 0.05$, **$p < 0.01$, ***$p < 0.001$, n.s.: not significant. Student's *t*-test.

Movie Legends

Supplementary Movie 1. Time-lapse movie of confocal images in empty vector (EV) RAW264.7 cells expressing GFP-tagged ST2L and RFP-tagged EV. Images were captured with confocal microscope at 488 and 561 nm laser every 1 s over a period of 3 min. Time intervals in minutes and seconds are shown. Stills corresponding to frames from 00:00 to 02:43 of this movie are presented in **Fig. 2F** (EV). Scale bars: 20 μm.

Supplementary Movie 2. Time-lapse movie of confocal images in Rab37 wild-type RAW264.7 cells expressing GFP-tagged ST2L and RFP-tagged Rab37. Images were captured with confocal microscope at 488 and 561 nm laser every 1 s over a period of 3 min. Time intervals in minutes and seconds are shown. Stills corresponding to frames from 00:00 to 02:43 of this movie are presented in **Fig. 2F** (Rab37). Scale bars: 20 μm.

Supplementary Table 1. The plasmids and their characteristics used in the current study.

Plasmid	Target	Insert (bp)	Function	Source
pcDNA3.1-V5/His-vector	None[a]	0	Vector control	Invitrogen
pcDNA3.1-V5/His-mRab37^{WT}	Mouse Rab37^{WT}	672	Overexpression	Homemade[b]
pcDNA3.1-V5/His-mRab37^{QL}	Mouse Rab37^{QL}	672	Overexpression	Homemade[b]
pcDNA3.1-V5/His-mRab37^{TN}	Mouse Rab37^{TN}	672	Overexpression	Homemade[b]
pDsRed2-C1-mRab37^{WT}	Mouse Rab37^{WT}	672	Overexpression	Homemade[c]
pLV-mIL1RL1-GFPSpark	Mouse IL1RL1[d]	2433	Overexpression	Sino Biological Inc
pGL4.17 vector	None[a]	0	Vector control	Promega
pGL4.17-ST2L promoter-WT	ST2L promoter	1631	Promoter activity	Homemade[e]
pGL4.17-ST2L promoter Mutant 1	ST2L promoter	1631	Promoter activity	Homemade[e]
pGL4.17-ST2L promoter Mutant 2	ST2L promoter	1631	Promoter activity	Homemade[e]
pGL4.17-ST2L promoter Mutant 1 & 2	ST2L promoter	1631	Promoter activity	Homemade[e]
shRNA-mIL-33-pLKO.1	Mouse IL-33	--[f]	Knockdown	Academia Sinica
shRNA-p65-pLKO.1	Human p65	--[f]	Knockdown	Academia Sinica

pEGFP-C1-p65	Human p65	372	Overexpression	Homemade[e]

[a] The plasmid is used as a backbone vector and therefore has no inserted fragment.

[b] Rab37-WT, Q89L, or T43N were PCR-amplified with designated mutation at the primer sequences and cloned into pcDNA3.1-V5/His expression vector to generate V5/His-tagged Rab37 expression vector.

[c] Mouse Rab37 cDNA was PCR-amplified and cloned into the pDsRed2-C1 expression vector to generate an RFP-tagged Rab37 expression vector.

[d] *IL1RL1* gene encodes ST2L.

[e] ST2L promoter-WT, Mutant 1, Mutant 2, Mutant 1 & 2 were PCR-amplified and cloned into pGL4.17 vector to generate ST2L promoter vector.

[f] shIL-33 lentiviral particles were produced in packaging vector pCMV-△R8.91 envelop plasmid (pMD.G) and targeted shRNA pLKO.1 plasmid. The viruses were obtained from the RNAi core (Academia Sinica, Taipei, Taiwan).

Supplementary Table 2. The primers used in the current study.

Gene	Primer	Sequences (5'→ 3')	Application[a]	PCR size (bp)	Tm (°C)
Mouse *IL1RL1 V1* mRNA[b]	Forward	GCCCGACGTTCTTGAAAATA	RT-qPCR	177	60
	Reverse	ATCTCCTGCTCGTAGGCAAA			
Mouse *IL1RL1 V123* mRNA[b]	Forward	GCGGAGAATGGAACCAACTA	RT-qPCR	158	60
	Reverse	AAGCAAGCTGAACAGGCAAT			
Human *IL1RL1 V1* mRNA[b]	Forward	TCACCAGATTCTGCCTGATG	RT-qPCR	154	60
	Reverse	GATCTGAGGGGTCAGGATGA			
Mouse *Rab37* mRNA	Forward	AACTACGATCTCACCGGCAA	RT-qPCR	248	60
	Reverse	AGCAAAGCCTGAGCATCTCG			
Mouse *TNF-α* mRNA	Forward	ATGAGCACAGAAAGCATGATC	RT-qPCR	276	60
	Reverse	TACAGGCTTGTCACTCGAATT			
Mouse *Fizz1* mRNA	Forward	CCTGCTGGATGACTGCTA	RT-qPCR	157	60
	Reverse	TGGGTTCTCCACCTCTTCAT			
Human *Fizz1* mRNA	Forward	CTGCTGGGATGGCTGTCACT	RT-qPCR	151	60
	Reverse	TCTCAGCCTCCTCCCTGTCA			
Mouse *Arg1* mRNA	Forward	CCACAGTCTGGCAGTTGGAAG	RT-qPCR	106	60
	Reverse	GGTTGTCAGGGGAGTGTTGATG			
Mouse *Ym1* mRNA	Forward	GCCACTGAGGTCTGGGATGC	RT-qPCR	113	60
	Reverse	TCCTTGAGCCACTGAGCCTTC			
Mouse *Actin* mRNA	Forward	GGTCCACACCCGCCACCAG	RT-qPCR	75	60
	Reverse	CACATGCCGGAGCCGTTGTC			

Gene	Primer	Sequences (5'→ 3')	Application[a]	PCR size (bp)	Tm (°C)
Human *Actin* mRNA	Forward	GGCGGCACCACCATGTACCCT	RT-qPCR	202	60
	Reverse	AGGGGCCGGACTCGTCATACT			
Human *ST2L* promoter site1	Forward	GAGTTTCGCTGTGTCACCAA	ChIP-PCR	217	60
	Reverse	CATGGTAGCTCACGCCTGTA			
Human *ST2L* promoter site2	Forward	TTGCTGCAGAGTGAGCTGAT	ChIP-PCR	231	60
	Reverse	GCAAAACCTCCCTAACACCA			
Human *ST2L* promoter WT	Forward	TAGGTACCTTTGTCTGTG TGTTGAGGTGGGGGTGGG	Promoter activity	1631	58
	Reverse	AGTGCTCGAGCTGCCCACAGT TTCACAACTCAGAAAGCCA			
Human *ST2L* promoter mutant 1	Forward	TAGAGACGGTGTTTCACCATGTTGGC	Promoter activity	1631	55
	Reverse	CCAACATGGTGAAACACCGTCTCTACT			
Human *ST2L* promoter mutant 2	Forward	TGTGACTATAGTGAAGTTACAACTACCAAC	Promoter activity	1631	55
	Reverse	GTTGGTAGTTGTAACTTCACTATAGTCACA			
Human p65	Forward	GGCCTTGCTTGGCAACAG	RT-qPCR	100	60
	Reverse	CACAGGTATGCCCTGGTTCA			

[a] RT-qPCR: Quantitative reverse-transcriptase polymerase chain reaction; ChIP: chromatin immunoprecipitation polymerase chain reaction.

[b] *IL1RL1* gene encodes ST2L.

Supplementary Table 3. The antibodies and their reaction condition used in the current study.

Target	kDa	Raised In	Application	Dilution	Source	Catalog No.
Rab37	27	Rabbit	Western blotting	1:1000	Proteintech	13051-1-AP
			Immunofluorescence	1:5000		
			Florescent immunohistochemistry	1:5000		
NFκB p65	65	Rabbit	Western blotting	1:1000	Merck	06-418
			Immunofluorescence	1:3000		
Phospho-NF-κB p65 (Ser536)	65	Rabbit	Western blotting	1:1000	Cell Signaling Technology	3031
			Immunofluorescence	1:3000		
			ChIP[b]	1:500		
β-actin	42	Mouse	Western blotting	1:5000	GeneTex	GTX26276
ST2	62	Mouse	Western blotting	1:1000	Leadgene Biomedical	LGT2105
			Immunofluorescence	1:1000		
			Immunofluorescence-immunohistochemistry (IHC)	1:1000		
	--[a]	Rat anti-Mouse	Flow cytometry (BV421)	1:200	BD Bioscience	566310
IL-33	18	Mouse	Western blotting	1:1000	Leadgene Biomedical	LGT3314
	--[a]		IHC	1:300		
GAPDH	37	Mouse	Western blotting	1:1000	Santa Cruz	Sc-32233
Calnexin	68	Rabbit	Western blotting	1:5000	Genetex	GTX109669
V5 tag	--[a]	Mouse	Immunoprecipitation	1:200	Invitrogen	46-0705
His tag	--[a]	Rabbit	Flow cytometry	1:200	Genetex	GTX115045

Target	kDa	Raised In	Application	Dilution	Source	Catalog No.
CD8	--[a]	Rabbit	Immunofluorescence-IHC	1:1000	Abcam	ab217344
PD-L1	--[a]	Rabbit	Immunofluorescence-IHC	1:1000	Cell Signaling Technology	13684
CD206	--[a]	Rabbit	Immunofluorescence-IHC	1:1000	Abcam	ab64693
	--[a]	Rat anti-Mouse	Flow cytometry (Alexa647)	1:200	BD Bioscience	565250
CD11b	--[a]	Rat anti-Mouse	Flow cytometry (BB515)	1:200	BD Bioscience	564454
CD86	--[a]	Rat anti-Mouse	Flow cytometry (BB605)	1:200	BD Bioscience	563055
CD4	--[a]	Rat anti-Mouse	Flow cytometry (PerCP)	1:200	BD Bioscience	561090
CD25	--[a]	Rat anti-Mouse	Flow cytometry (PE)	1:200	BD Bioscience	562695
Foxp3	--[a]	Rabbit	Immunofluorescence-IHC	1:1000	Genetex	GTX107737
	--[a]	Rat anti-Mouse	Flow cytometry (BV421)	1:200	BD Bioscience	562996
DAPI	--[a]	--[c]	Immunofluorescence	--[c]	Genetex	GTX30920
Opal 520 Reagent	--[a]	--[c]	Immunofluorescence-IHC	1:100	Akoya Biosciences	FP1013
Opal 570 Reagent	--[a]	--[c]	Immunofluorescence-IHC	1:100	Akoya Biosciences	FP1014
Opal 620 Reagent	--[a]	--[c]	Immunofluorescence-IHC	1:100	Akoya Biosciences	FP1495A

[a] Molecular weight is not applicable to this antibody in such an application.

[b] ChIP: Chromatin immunoprecipitation.

[c] DAPI is a commercial product for nuclear staining and Opal reagents are commercial products for immunofluorescence-IHC.
