# Supplementary figures and images for: IL-33/NF-κB/ST2L/Rab37 positive-feedback loop promotes M2 macrophage to limit chemotherapeutic efficacy in lung cancer
Source: Cell Death Dis. 2024 May 22;15(5):356. doi: 10.1038/s41419-024-06746-y (PMC11111460; doi:10.1038/s41419-024-06746-y)

Fig 2B.

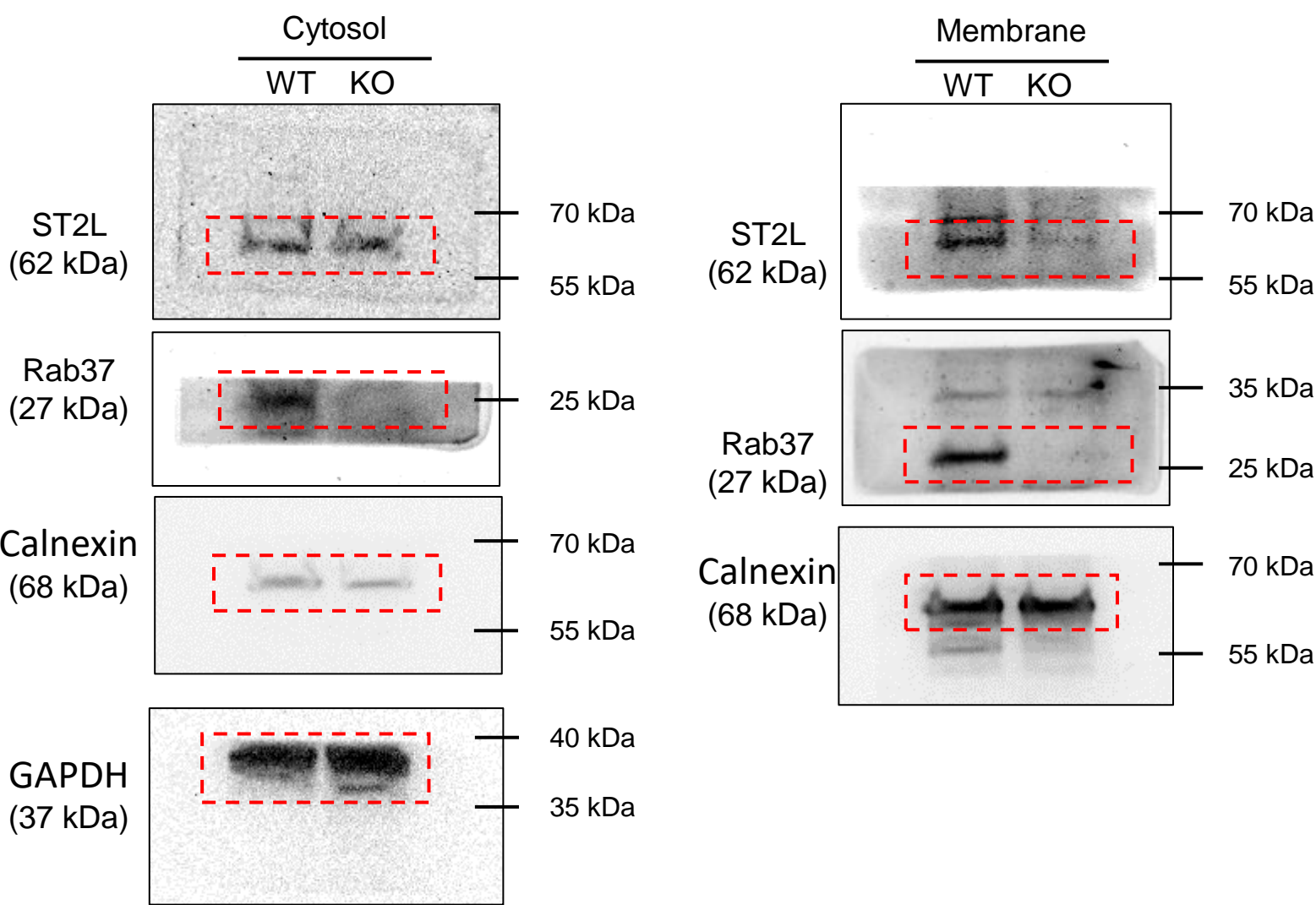

Fig 2I.

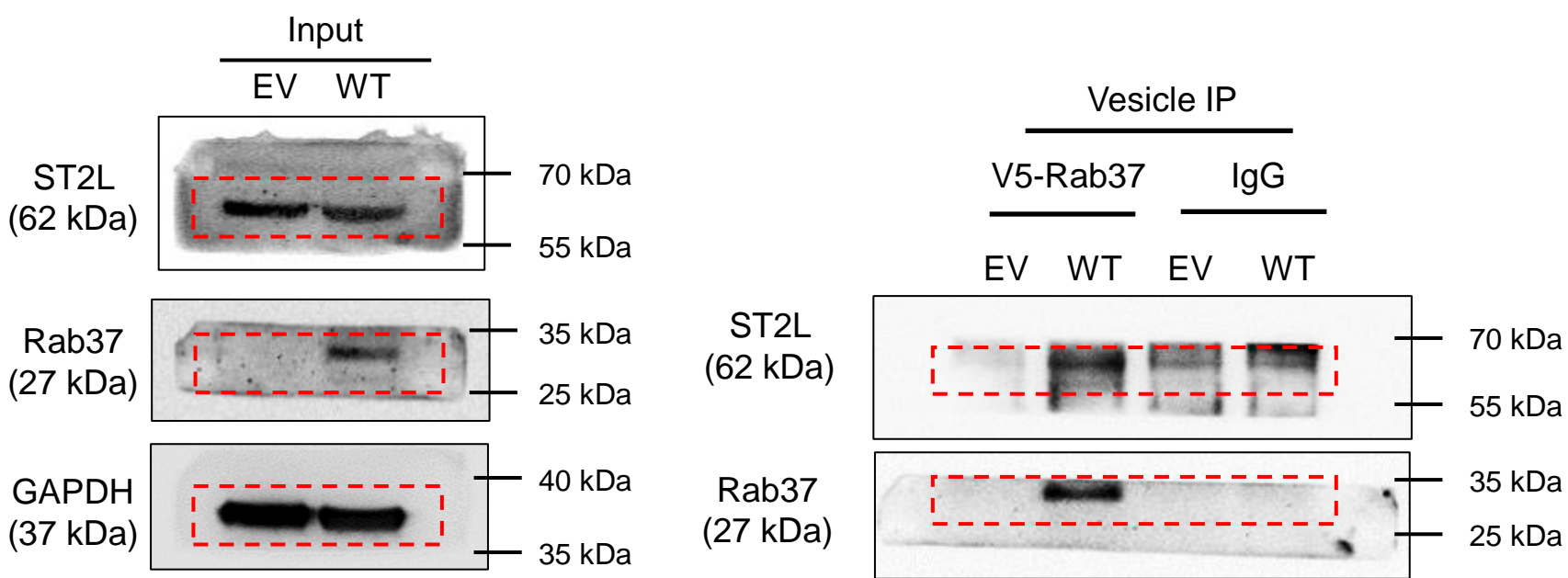

Fig 4B.

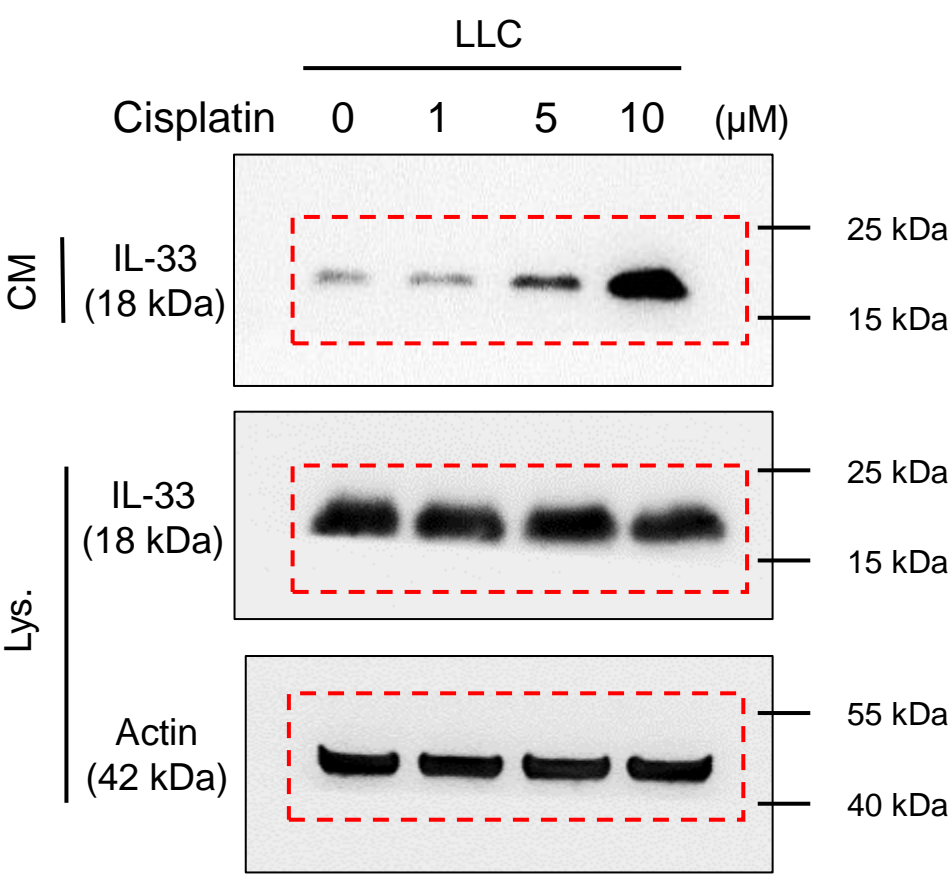

Fig S2F.

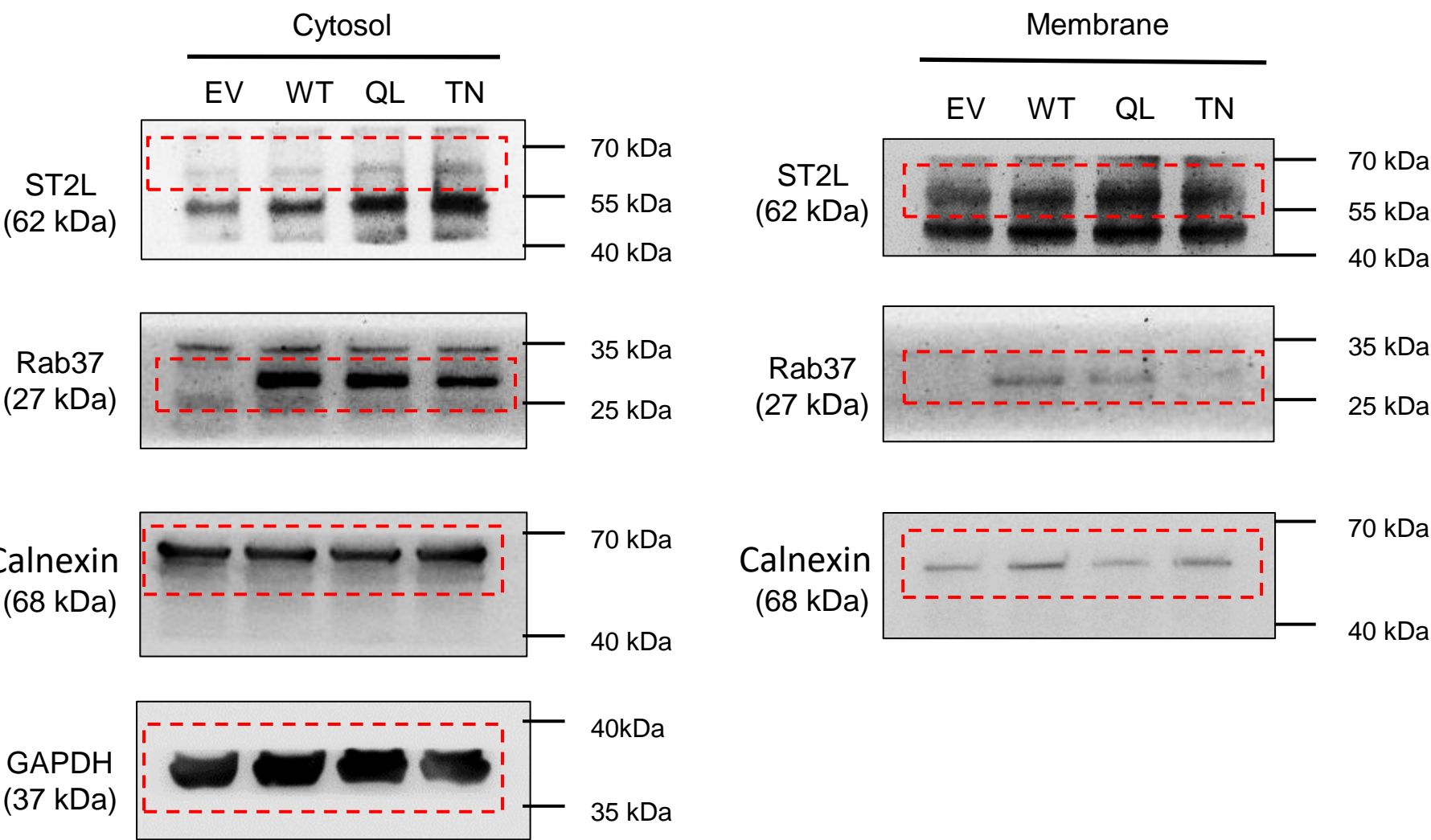

Fig S3C.

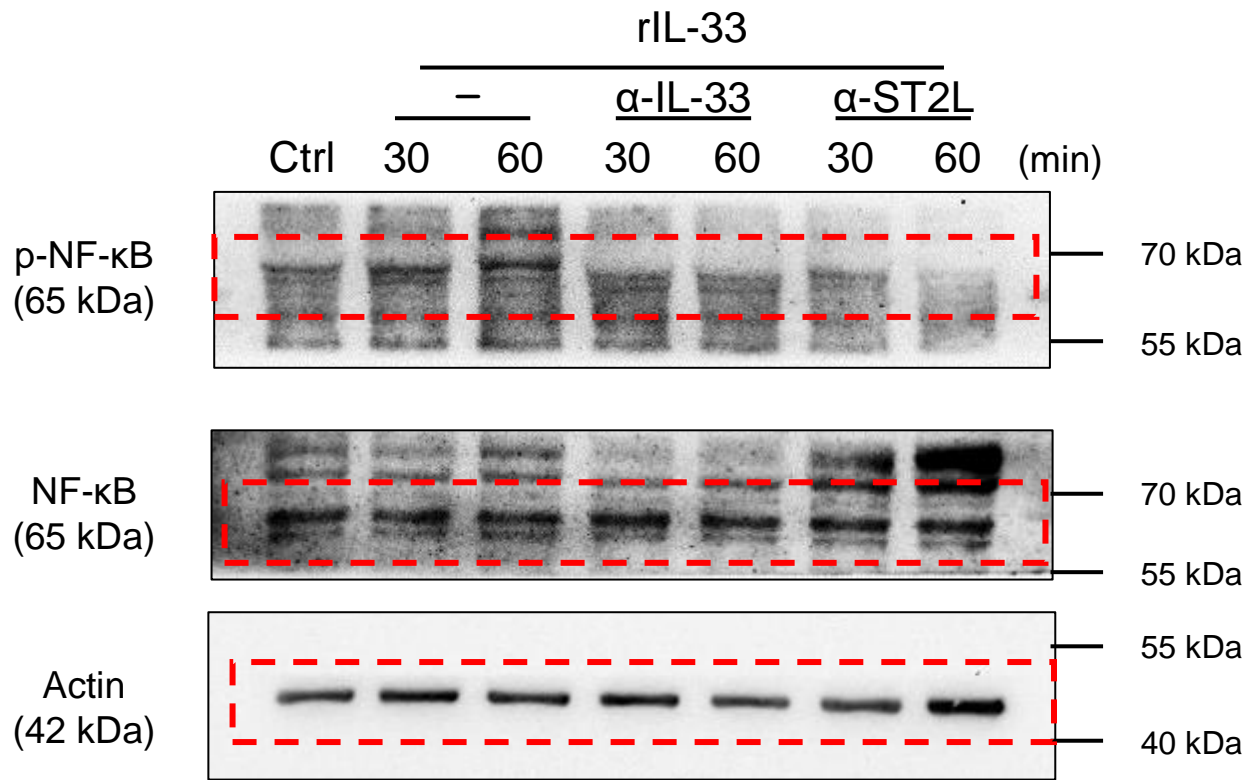

Fig S4A.

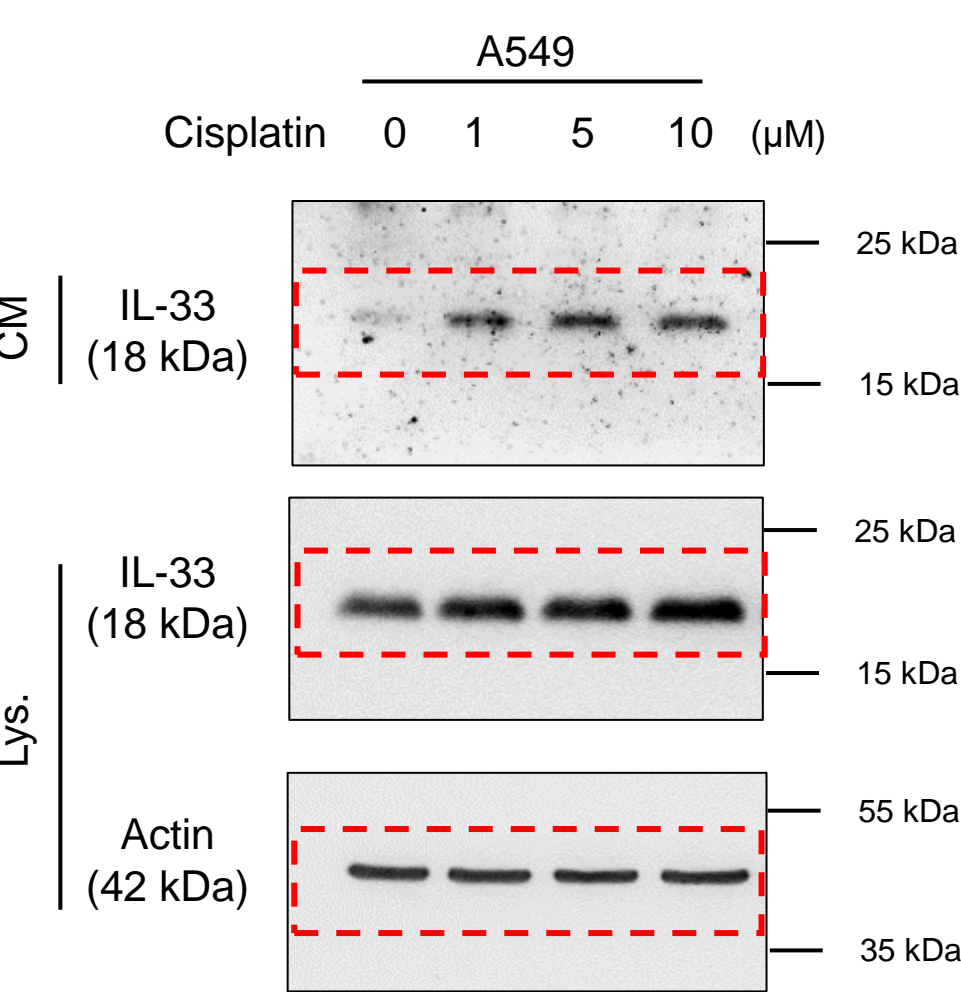

Fig S4C.

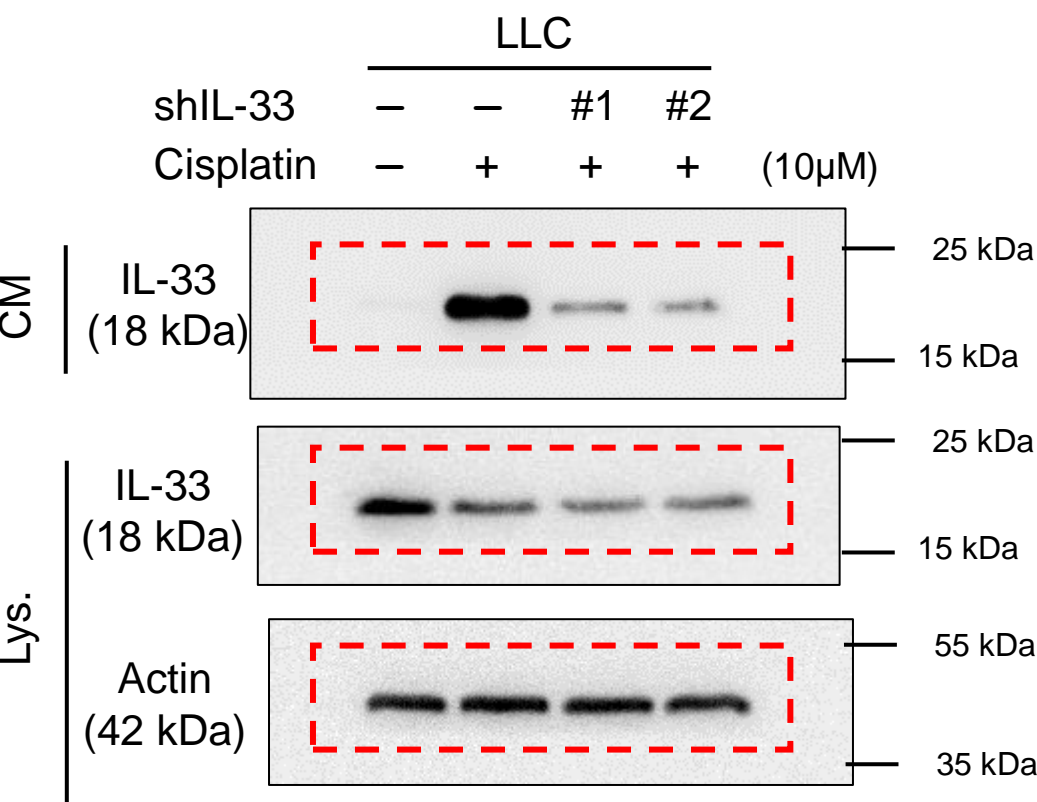

Supplement: Supplementary file 4 — Uncropped Western Blots [file 41419_2024_6746_MOESM4_ESM.pdf]
